# Improved slime mould algorithm based on hybrid strategy optimization of Cauchy mutation and simulated annealing

**DOI:** 10.1371/journal.pone.0280512

**Published:** 2023-01-25

**Authors:** Xiaoyi Zhang, Qixuan Liu, Xinyao Bai

**Affiliations:** 1 School of Biological and Agricultural Engineering, Jilin University, Changchun, Jilin Province, China; 2 Key Laboratory of Bionic Engineering, Ministry of Education, Jilin University, Changchun, Jilin Province, China; 3 Economic Information Center of Jilin Province, Changchun, Jilin Province, China; Torrens University Australia, AUSTRALIA

## Abstract

In this article, an improved slime mould algorithm (SMA-CSA) is proposed for solving global optimization and the capacitated vehicle routing problem (CVRP). This improvement is based on the mixed-strategy optimization of Cauchy mutation and simulated annealing to alleviate the lack of global optimization capability of the SMA. By introducing the Cauchy mutation strategy, the optimal solution is perturbed to increase the probability of escaping from the local extreme value; in addition, the annealing strategy is introduced, and the Metropolis sampling criterion is used as the acceptance criterion to expand the global search space to enhance the exploration phase to achieve optimal solutions. The performance of the proposed SMA-CSA algorithm is evaluated using the CEC 2013 benchmark functions and the capacitated vehicle routing problem. In all experiments, SMA-CSA is compared with ten other state-of-the-art metaheuristics. The results are also analyzed by Friedman and the Wilcoxon rank-sum test. The experimental results and statistical tests demonstrate that the SMA-CSA algorithm is very competitive and often superior compared to the algorithms used in the experiments. The results of the proposed algorithm on the capacitated vehicle routing problem demonstrate its efficiency and discrete solving ability.

## Introduction

Establishing a mathematical model to solve practical problems and realizing faster and better solutions to the model is one of the goals pursued by current academic research, and the way to achieve it depends on intelligent optimization algorithms with different strategies. The solution performance of intelligent optimization algorithms under different optimization strategies is different [1]. The intelligent optimization algorithm is a kind of optimization method based on mathematics that is generated by simulating the behavior of natural biological clusters or natural phenomena and is used to solve various practical optimization problems. It is widely used in signal processing, production scheduling, medical applications, image processing, path planning, and other fields [2].

Throughout the development of intelligent optimization algorithms, the shortfalls of classical optimization strategies have been the starting point for the creation of new algorithms, such as the time-out of Newton’s method in the face of complex mathematical processes, so people are paying attention to optimization algorithms inspired by nature. For examples, see the genetic algorithm (GA) [3], the differential evolution algorithm (DE) [4], the immune algorithm (IA) [5], the ant colony algorithm (ACO) [6], the particle swarm algorithm (PSO) [7], the simulated annealing algorithm (SA) [8], etc. In addition, due to the increase in the actual needs of the current society and the improvement of computer computing power, in order to improve the accuracy of the solution, more and more scholars are committed to developing new algorithms based on the existing algorithm solution strategies and extending them to many problems in multidisciplinary optimization. Some of these methods are described below: Water Strider Algorithm (WSA) [9], Fitness-Distance Balance (FDB) [10], Hybrid Invasive Weed Optimization-Shuffled Frog-Leaping Algorithm (SFLA-IWO) [11], Algorithm of the Innovative Gunner (AIG) [12], Red Deer Algorithm (RDA) [13], and Dragonfly Algorithm (DA) [14], Whale Optimization Algorithm (WOA) [15], Harry Hawk Algorithm (HHO) [16], Gray Wolf Optimization Algorithm (GWO) [17], and Slime Mold Algorithm (SMA) [18], Mountain Gazelle Optimizer (MGO) [19], Starling Murmuration Optimizer (SMO) [20], Stochastic Paint Optimizer (SPO) [21], etc., and these algorithms are also satisfactory in the existing practical problem tests.

It is precisely because the slime mold algorithm (SMA) has the advantages of a simple structure, high applicability, and strong optimization ability that the improvement of the slime mold algorithm and its application to the capacitated vehicle routing problems are the main research contents of this paper. The original SMA is a new algorithm proposed by simulating the foraging process of slime mold, the changes in the vein structure of slime mold, and the three forms formed by positive feedback and negative feedback generated by biological oscillators [18]. But unfortunately, since the slime mould algorithm has the defect that it is easy to fall into the local optimal solution in the process of finding the optimal solution, how can we develop or improve a new algorithm to make it faster and more accurate to obtain the global optimal solution has become one of the goals pursued by researchers [22]. The main purpose of this paper is to optimize and improve the slime mould algorithm by introducing the annealing operator(SA) and the Cauchy mutation strategy (CM), so as to ensure that the improved algorithm has greater population diversity in the local optimization and can escape the local extreme value smoothly. At the same time, accelerate the convergence speed at which the population approaches the optimal position and enhance the solving ability and accuracy of its global optimal solution.

The main contributions of this study are as follows:

1. The global optimization accuracy of the slime mould algorithm is improved, the annealing link is introduced into the process of updating the position of the slime mould algorithm, and the Cauchy mutation strategy is used to perturb the optimal local solution to reduce the probability of falling into the optimal local solution, and then An improved slime mould algorithm (SMA-CSA) based on mixed strategy optimization of Cauchy mutation and simulated annealing is proposed.
2. The paper employs the CEC2013 standard functions and the Wilcoxon rank-sum test to compare the development and exploration performance differences between SMA, SMA-CSA, and other comparative algorithms.
3. The proponents of the slime mould algorithm and many improvers lack the solution verification of discrete problems such as the vehicle routing problem with capacity constraints. Therefore, this paper applies SMA and SMA-CSA to two CVRP standard example packages, respectively, and further analyzes the experimental results to obtain the performance difference under different optimization strategies and the supplementary research of the algorithm on such problems.

The rest of the paper is organized as follows: Section II presents existing strategies for improving the slime mould algorithm and application areas. The third section introduces the mathematical description of the slime mould algorithm, as well as the improvement ideas for the slime mould algorithm, the description and expression of the mathematical formula, and the pseudo-code of the improved algorithm. In the fourth section, the CEC2013 and CVRP examples are simulated for SMA and SMA-CSA, and the differences between the calculation results and the comparison algorithms are tested. Finally, Section V introduces the main conclusions and limitations of this study.

## Related work

### Improvement of slime mould algorithm

Previous studies have fully demonstrated the superiority of metaheuristic optimization algorithms in solving large-scale search and optimization problems [23]. In the last year of research, the slime mold algorithm has fully demonstrated its applicability in engineering optimization problems as a new meta-heuristic optimization method. However, in the in-depth understanding of the slime mould algorithm, it is found that it has the defects of slow convergence speed, weak global search ability, and easy falling into the optimal local solution. Moreover, when solving specific practical optimization problems, the improvement of the optimal results of the slime mould algorithm is still limited, so how to improve the performance of SMA has become a direction of future research. Table 1 lists the existing optimization ideas for the SMA algorithm.

**Table 1:** A summary of some improved variants of the slime mold algorithm, including their names, improvement ideas, and the source of the algorithms.

	Intelligent optimization algorithm	Improvement ideas	Sources
Adaptive Optimization	Adaptive opposition slime mould algorithm (AOSMA)	An adaptive method to decide whether to use opposition-based learning (OBL). Sometimes OBL is used to further increase exploration. Furthermore, it maximizes utilization by replacing one random search agent with the best one in location updates	Naik, 2021
	Adaptive slime mould algorithm (ASMA)	Introduce an adaptive selection mechanism to achieve control over the appropriate parameters of the original SMA, and introduce an opposition‐based learning operator to improve the optimal solution.	Lin, 2022
	Dominant Swarm with Adaptive T-distribution Mutation-based Slime Mould Algorithm (DTSMA)	Using an adaptive t-distribution mutation balances to enhance the exploration and exploitation ability of the original SMA, and a new exploitation mechanism is hybridized to increase the diversity of populations, forming Dominant Swarm with Adaptive T-distribution Mutation-based Slime Mould Algorithm (DTSMA).	Yin, 2022
Chaotic Optimization	Chaotic slime mould optimization algorithm (CSMA)	Apply ten different chaotic maps to generate chaotic values to replace random values in SMA. Using a Chaos Map aims to increase the speed at which the SMA converges globally and prevent it from falling into a local solution.	Altay, 2022
	Chaos-opposition-enhanced slime mould algorithm (CO-SMA)	The Chaotic Search Strategy (CSS) and crossover-opposition strategy (COS) are introduced to the original SMA to improve the global search performance.	Rizk-Allah, 2022
	Chaotic slime mould algorithm (CSMA)	The chaotic search strategy is introduced to replace the random value of the original SMA. Moreover, the ergodicity of the chaotic value is used to replace the randomness, which is used to jump out of the optimal local solution.	Singh, 2022; Abid, 2022; Chen, 2020
Hybrid Optimization	Hybrid SMA-WOA (HSMA-WOA)	Apply the WOA algorithm within the first half of the iteration and use its exploration capabilities to explore the search space. After reaching a certain value, WOA will stop. Subsequently, HSMA-WOA started using SMA to search for a better solution.	Abdel-Basset, 2020;
	Hybrid improved slime mould algorithm with adaptive β hill climbing	Brownian motion and tournament selection mechanisms are introduced into the original SMA to improve the exploration ability. In addition, the adaptive β-hill climbing and AβHC local search algorithms are introduced to form the Hybrid improved slime mould algorithm with adaptive β-hill climbing.	Sun, 2020
	SMA combined to Adaptive Guided Differential Evolution Algorithm (AGDE) (SMA-AGDE)	A solution for updating the SMA using AGDE’s mutation and crossover process. Use the gradient method to escape subregions before updating the solution. Component notation (r−CR) controls exploration and development direction.	Houssein, 2021
	Improved Slime Mould Algorithm (ISMA)	Two equations were borrowed from the Sine Cosine Algorithm (SCA) to update the position of the solution to obtain the best solution, forming the Improved Slime Mould Algorithm.	Hassan, 2021
	A hybrid Slime mould algorithm with whale optimization algorithm	Borrowing the position updating behaviour of the Whale Optimization Algorithm (WOA) to enhance the searching behaviour of the slime mould algorithm (SMA).	Bhandakkar, 2022

Like other metaheuristic algorithms, solving the balance between exploration and development in SMA can effectively improve the global search performance of the algorithm. Therefore, methods such as self-adaptive improvement of fixed parameters or the introduction of self-adaptive weight factors to balance the development and exploration performance of SMA have gradually become effective measures for many researchers. For example, ASMA [24] is proposed by adopting a suitable mechanism for adaptively selecting SMA control parameters and introducing an adversarial learning operator; DTSMA [25] uses adaptive t-distribution variation balance to enhance the ability to explore and exploit; and AOSMA uses an adaptive approach to decide whether opposition-based learning (OBL) will be used or not [26]. Moreover, from the experimental results, it can be seen that the global optimization results have significantly improved with the adaptive optimization of the slime mould algorithm compared with the original algorithm.

Similar to the adaptive improvement algorithm, many researchers also tend to use the chaotic map to optimize and improve the meta-heuristic optimization algorithm, and past research has confirmed that the use of the chaotic map can improve the solution performance of the optimization algorithm. The chaotic SMA (CSMA) proposed by reference [27] also confirmed the applicability of the chaotic mapping method to the performance improvement of the SMA algorithm in the test of 62 benchmark functions. In addition, other scholars have achieved similar results by improving SMA based on chaos mappings, such as the chaos-opposition-enhanced slime mould algorithm (CO-SMA) [28] and the Chaotic slime mould algorithm (CSMA) [29–31].

In addition, borrowing the traditional algorithm position update strategy to optimize the original SMA algorithm and improve the solution performance of the original algorithm is also one of many research schemes. For example, borrowing the location update behavior of the Whale Optimization Algorithm (WOA) can enhance the global search behavior of the original slime mould algorithm [32, 33]. Similarly, the two equations of the Sine-Cosine Algorithm (SCA) borrowed by reference [34] to update the optimal solution position also achieved similar improvements. In addition, the introduction of the adaptive β-hill-climbing algorithm mechanism has also been proved to have the ability to strengthen the local search of SMA, which can well balance the algorithm development and exploration performance [35]. Some researchers have integrated and optimized the differential evolution algorithm and the slime mould algorithm, and the solution ability of the resulting hybrid algorithm has also been greatly improved [36, 37].

### Application of slime mould algorithm

By reviewing the literature, it appears that the current slime mould algorithm is widely used. Researchers have applied the slime mould algorithm and its variants to engineering optimization problems and other research fields. For example, solving single- and du-al-objective economic and emission scheduling (EED) problems considering valve point effects [34]; determining the best operating rules for complex hydropower multi-reservoir prediction problems [38]; distributed generation (DG) solution of distribution network reconfiguration (DNR) problem [39]; photovoltaic model optimization design (Lin, 2022); demand estimation of urban water resources problem [40]; feature selection [41]; Reliability optimization of micro-milling cutting parameters [42]; Opti-mal Power Flow Problem [43]; A Cost-Effective Solution for Non-Convex Economic Load Dispatch Problems in Power Systems [44]; path planning and obstacle avoidance problem in mobile robots [45], optimal load-shedding in distribution system problem [30], etc.

Referring to the application scenarios of other optimization algorithms, the slime mold algorithm can also do a lot. For example, an improved whale optimization algorithm for COVID-19 medical feature selection [46]; a dynamic arithmetic optimization algorithm for Tesla optimization under natural frequency constraints [47]; Dynamic Water Strider Algorithm for optimal design of bone structure [48]; A Quantum-Based Avian Navigation Optimizer for feature selection of high-dimensional medical data [49]; Multi-trial vector differential evolution algorithm for non-decomposition large-scale global optimization [50]; An Advanced Dipper-Throated Meta-Heuristic Optimization Algorithm for Digital Image Watermarking [51]; A Multi-Search Arithmetic Optimization Algorithm for Efficient Text Document Clustering [52]; An Effective Whale Optimization Algorithm to Solve the Optimal Power Flow Problem [53], etc.

It can be seen from the literature results that the slime mould algorithm has applicability in solving optimization problems, and with the improvement of the original algorithm, the solution accuracy and convergence speed of the improved slime mould algorithm have been improved. Among the many problems solved, there is still a lack of verification solutions for vehicle routing problems such as CVRP, VRPTD, VRPTW, and GVRP, as well as discrete problems such as warehouse routing problems (LRP). Therefore, in the fourth section, this paper solves the CVRP problem discretely and uses two CVRP calculation examples to expand the application research of the slime mould algorithm and its variants.

## Methodology

Because the original slime mould algorithm is easy to fall into the optimal local solution; therefore, in the algorithm design process, we need to introduce a new mechanism to improve the global optimization ability of the original slime mould algorithm and S1 Algorithm shows the pseudo-code of SMA.

### Slime mould algorithm

#### Approach food

**Fig 1:**
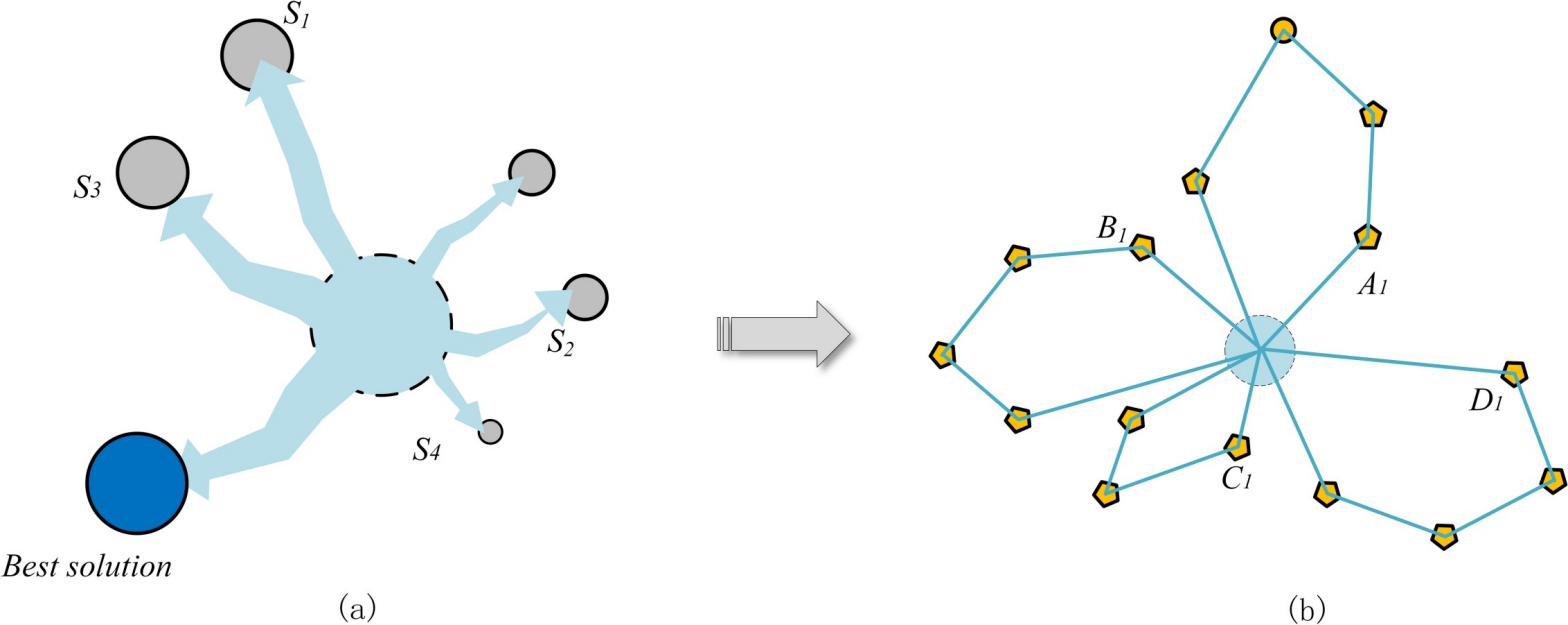
Slime mold search optimal solution and the CVRP solution path. The nodes in the graph represent the possible optimal solutions in the slime mould search process; the nodes in the b graph represent the client nodes in the CVRP problem.


The slime mould spontaneously approaches the food according to the smell of the food, and the following mathematical expression can express its expansion pattern. Fig 1 shows how the slime mold search searches for the optimal solution and the solution to the CVRP problem.

$$\vec{X\left(t+1\right)}=\left\{\begin{array}{cc} \vec{X_{b}\left(t\right)}+\vec{vb}\cdot \left(\vec{W}\cdot \vec{X_{A}\left(t\right)}-\vec{X_{B}\left(t\right)}\right) & ,r<p\\ \vec{vc}\cdot \vec{X\left(t\right)} & ,\;r\geq p \end{array}\right.$$ (1)

$$\vec{vb}=\left[-a,a\right]$$ (2)

Where $\vec{vb}$ is a parameter in the range of [-a, a], and the formula is shown in Eq (2); where the value of t represents the current number of iterations, and max_*t* represents the maximum iterations as shown in Eq (3); $\vec{vc}$ is a linear decreasing process, and the absolute value takes values from 1 to 0; $\vec{X_{b}}$ represents the current individual location with the highest food odour; $\vec{X}$ represents the position of slime mould; $\vec{X_{A}}$ and $\vec{X_{B}}$ represent two individuals randomly selected from all slime mould; $\vec{W}$ represents the specific weight of slime mould.


$$a=arctan\;h\left(-\left(\frac{t}{max\_t}\right)+1\right)$$ (3)

p=tanh|S(i)−DF| (4)

$$\vec{W\left(smell\ln dex\left(i\right)\right)}=\left\{\begin{array}{c} 1+r\cdot log\left(\frac{bF-S\left(i\right)}{bF-wF}+1\right),condition\\ 1-r\cdot log\left(\frac{bF-S\left(i\right)}{bF-wF}+1\right),others \end{array}\right.$$ (5)

$$Smell\ln dex=sort\left(S\right)$$ (6)

The Eq (4) denote the P-value, where *S*(*i*) represents the fitness of the mucilaginous individual, *i* ∈ (1,2,3,……, *n*); *DF* represents the best fitness obtained by the slime mould individual in all iterations. The Eq (5) simulates the relationship between food odour concentration and mucor vein width; where the condition represents the *S*(*i*) value in the first half of the optimal solution ordering, this condition serves to simulate the search pattern chosen by the mucor based on food concentration; the *r* is used to simulate the uncertainty in the form of mucor vein contraction, and the value is a random value in the interval [0,1]; the log contributes to reduce the rate of change of the numerical contraction. The *bF* value represents the optimal iteration value in the current iteration process; *wF* value is the worst value in the current iteration process; *Smelllndex* is the ranking of all iterations.

#### Wrap food

The Eq (7) simulates the process of slime mould searching for food and updating its position after searching for food based on Eq (1) and combines Eq (5) to simulate the shrinkage of the tissue structure of slime mould veins. In the process of searching for food, the slime mould determines the search weight of the area by judging the concentration of the food odour between the areas. The higher the concentration of food, the stronger the bio-vibration of the bio-vibrator, the faster the flow of cytoplasm, and the thicker the intercellular venous route, the greater the search weight of the area; otherwise, it will turn to search other areas.

$$\vec{X\left(t+1\right)}=\left\{\begin{array}{cc} rand\cdot \left(UB-LB\right)+LB, & rand<z\\ \vec{X_{b}\left(t\right)}+\vec{vb}\cdot \left(\vec{W}\cdot \vec{X_{A}\left(t\right)}-\vec{X_{B}\left(t\right)}\right), & r<p\\ \vec{vc}\cdot \vec{X\left(t\right)}, & r\geq p \end{array}\right.$$ (7)

Where *LB* and *UB* represent the size of the upper and lower boundaries of the slime search process; rand denotes a random value in the interval [0,1], and how to determine the parameter *z* value need to be discussed according to specific experiments.

#### Oscillation

The slime mold controls the thickness of their own venous network by their own oscillation fluctuations, the simulation process is shown in Eq (5). And the exploration process is realized by changing parameters such as $\vec{W}$, $\vec{vb}$, *$\vec{vc}$* according to the size of food concentration.

The values of $\vec{vb}$ and *$\vec{vc}$* oscillate randomly between the intervals [-a, a] and [–1,1], and eventually converge to zero. In addition, when the slime mold explores the food based on the food concentration, some of the slime will still be separated to explore other unknown areas. It is worth mentioning that the harsh environments encountered during exploration are informative for simulating real situations, such as strong sunlight and dryness.

### Proposed SMA-CSA

#### Cauchy mutation strategy

In the original slime mould algorithm, the generation of the optimal position depends on the concentration of food odor when the slime mould searches the unknown area. When the population of individuals tends to aggregate due to the increasing number of iterations, the optimal individual will lack the ability to quickly jump out of the optimal local solution, which makes the algorithm prone to the premature phenomenon. Therefore, to strengthen the ability of SMA to jump out of the optimal local solution, this paper introduces the fusion Cauchy mutation strategy to perturb the current optimal solution to ensure that the algorithm has greater population diversity during local optimization.

The Cauchy variation originates from the Cauchy distribution. According to the Cauchy distribution mechanism, the two ends of the Cauchy distribution function curve are longer, indicating that it can make it easier for individuals to escape from local extremes, and a smaller peak will guide individuals to spend less time searching for the optimal location. Therefore, the Cauchy mutation operator is introduced into the slime mould algorithm to fully use its strong perturbation ability to control the current optimal solution. The formula is as follows:

$$X_{best}^{*}\left(t+1\right)=X_{best}\left(t\right)+X_{best}\left(t\right)\times Cauchy\left(0,1\right)$$ (8)

$$X_{best}\left(t+1\right)=\left\{\begin{array}{cc} X_{best}\left(t\right)+X_{best}\left(t\right)\times Cauchy\left(0,1\right), & rand1\;>\;0.5\\ X_{best}\left(t\right), & rand1\;<\;0.5 \end{array}\right.$$ (9)

Among them, rand1 is a random probability number that obeys the normal distribution. When rand1>0.5, the algorithm uses the Cauchy operator to mutate the optimal solution, and its powerful perturbation ability can greatly improve the diversity of the population around the optimal solution. When the optimal individual of the local extreme value is found, it can help it escape quickly to ensure the robust optimization of the algorithm; when rand1<0.5, the algorithm retains the current optimal solution.

#### Annealing mechanism

The SMA-CSA algorithm proposed in this section is derived from the original SMA, and S2 Algorithm shows the pseudo-code of SMA-CSA. The annealing mechanism is introduced into the original SMA to improve the algorithm’s search and global optimization capabilities, the Metropolis sampling criteria as acceptance criteria, and control the annealing rate and minimum temperature, which form the algorithm’s structure in this paper. Compared with SMA, adding the annealing operator strengthens the robustness of the algorithm and reduces the probability of the algorithm falling into a local optimal solution during the search process. This method has also been extensively validated in solving problems such as TSP problems and 3D/2D fixed-outline floor planning [54, 55].

In SMA-CSA, whether to accept the current solution is determined by Eq (10):

$$PA\left(a\right)=e^{-F\left(a\right)/T_{c}}$$ (10)

$$T_{n}=T_{c}\times \varepsilon$$ (11)

$$T_{n}=\left(T_{p}-T_{c}\right)\times \omega +T_{c}$$ (12)

In Eq (10), *Tc* represents the current temperature, and the Metropolis sampling criterion *PA*(*a*) represents the possible probability that solution a is accepted. Each time a new solution is constructed, the current temperature is updated with Eq (11); where *ε* is a parameter that controls the rate of decrease of the current temperature, and *T*_*n*_ represents the temperature of the next iteration. It should be noted that in order to prevent the temperature from converging too fast to affect the quality of the solution, we need to introduce Eq (12) to adjust the current temperature; in Eq (12), *T*_*p*_ denotes the temperature at the last time when a feasible solution generated by SMA is accepted, where ω is a control parameter for the convergence rate, which is set to 0.998 in this paper.

**Fig 2:**
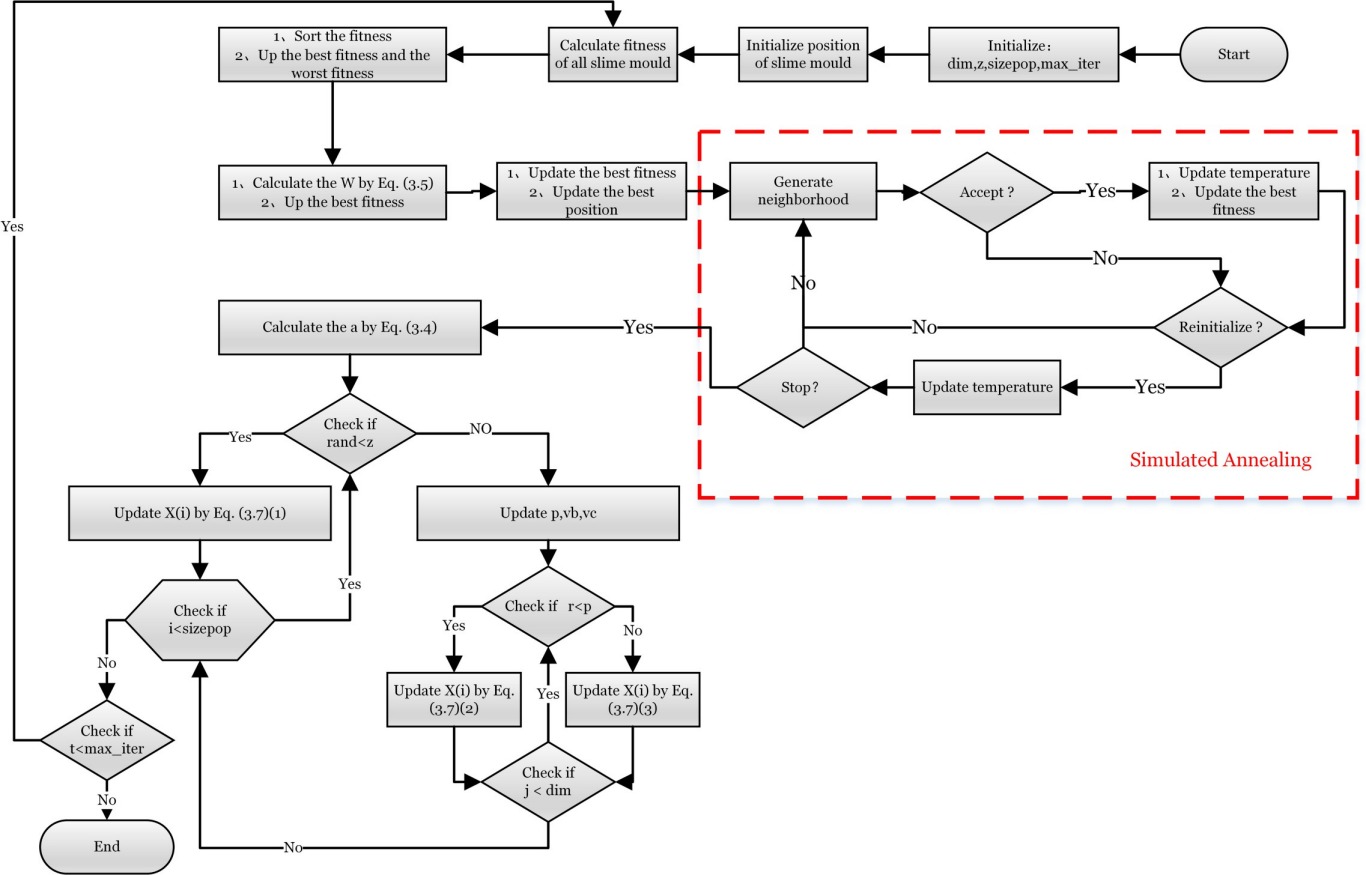
Flowchart of SMA-CSA. The annealing mechanism is in the red dotted box.

The improved algorithm structure and steps are shown in Fig 2.

## Simulation results

### CEC2013 benchmark functions

In this section, SMA-CSA is tested and compared with other algorithms using 23 standard test functions (Tables 2 and 3) from the CEC 2013. The average and standard deviation (STD) of the fitness function values are used as evaluation metrics to compare the algorithms’ merits. The description of the parameters for the CEC2013 benchmark algorithm is presented in Tables 2 and 3. F1-F7 are single-modal benchmark functions, which can effectively test the convergence rate and local search ability of the algorithm and have only one global optimal solution; F8-F13 are multimodal benchmark functions with multiple locally optimal solutions and one optimal global solution, which can effectively test the global search ability of the algorithm; and F14-F23 are composite benchmark functions, which can effectively test the algorithm exploitation ability and the balance search between the performance of the algorithm.

**Table 2:** Single-modal benchmark functions.

	Functions	dim	range
1	$f_{1}\left(x\right)=\sum_{i=1}^{n}x_{i}^{2}$	30	[-100,100]
2	$f_{2}\left(x\right)=\sum_{i=1}^{n}\left|x_{i}\right|+\prod_{i=1}^{n}\left|x_{i}\right|$	30	[-10,10]
3	$f_{3}\left(x\right)=\sum_{i=1}^{n}{\left(\sum_{j-1}^{i}x_{j}\right)}^{2}$	30	[-100,100]
4	$f_{4}\left(x\right)=max_{i}\left\{\left|x_{i}\right|,1\leq i\leq n\right\}$	30	[-100,100]
5	$f_{5}\left(x\right)=\sum_{i=1}^{n-1}\left[100{\left(x_{i+1}-x_{i}^{2}\right)}^{2}+{\left(-1\right)}^{2}\right]$	30	[-30,30]
6	$f_{6}\left(x\right)=\sum_{i=1}^{n}{\left(\left[x_{i}+0.5\right]\right)}^{2}$	30	[-100,100]
7	$f_{7}\left(x\right)=\sum_{i=1}^{n}ix_{i}^{4}+random\left[0,1\right]$	30	[-128,128]

**Table 3:** Multimodal benchmark function.

Name	Functions	dim	range
8	$minf_{8}\left(x\right)=\sum_{i=1}^{n}-x_{i}sin\left(\sqrt{\left|x_{i}\right|}\right)$	n	[-500,500]
9	$minf_{9}\left(x\right)=\sum_{i=1}^{n}\left[x_{i}^{2}-10\text{cos}\left(2\pi x_{i}\right)+10\right]$	n	[-5.12,5.12]
10	$minf_{10}\left(x\right)=-20\exp\left(-0.2{\left(\frac{1}{n}\sum_{i=1}^{n}x_{i}^{2}\right)}^{0.5}\right)-\text{exp}(\frac{1}{n}\sum_{i=1}^{n}\text{cos}\left(2\pi x_{i}\right))+20+e$	n	[-32,32]
11	$minf_{11}\left(x\right)=\frac{1}{4000}\sum_{i=1}^{n}x_{i}^{2}-\prod_{i=1}^{n}\text{cos}\left(\frac{x_{i}}{\sqrt{i}}\right)+1$	n	[-600,600]
12	$minf_{12}\left(x\right)=\frac{\pi }{n}\left\{10sin\left(\pi y_{1}\right)+\sum_{i=1}^{n-1}{\left(y_{i}-1\right)}^{2}\left[1+10sin^{2}\left(\pi y_{i+1}\right)\right]+{\left(y_{n}-1\right)}^{2}\right\}+\sum_{i=1}^{n}u\left(x_{i},10,100,4\right),y_{i}=1+\frac{x_{i}+1}{4}$ $u\left(x_{i},a,k,m\right)=\left\{\begin{array}{c} k{\left(x_{i}-a\right)}^{m}x_{i}>a\\ 0-a<x_{i}<a\\ k{\left(-x_{i}-a\right)}^{m}x_{i}<a \end{array}\right.$	n	[-50,50]
13	$minf_{13}\left(x\right)=0.1\left\{sin^{2}\left(3\pi x_{1}\right)+\sum_{i=1}^{n}{\left(x_{i}-1\right)}^{2}\left[1+sin^{2}\left(3\pi x_{i}+1\right)\right]+{\left(x_{n}-1\right)}^{2}\left[1+sin^{2}\left(2\pi x_{n}\right)\right]\right\}+\sum_{i=1}^{n}u\left(x_{i},5,100,4\right)$	n	[-50,50]
14	$F_{14}\left(x\right)={\left(\frac{1}{500}+\overset{25}{\underset{j=1}{\sum }}\frac{1}{j+\sum_{i=1}^{2}{\left(x_{i}-a_{ij}\right)}^{6}}\right)}^{-1}$	2	[-65,65]
15	$F_{15}\left(x\right)=\overset{11}{\underset{i=1}{\sum }}{\left[a_{i}-\frac{x_{1}\left(b_{i}^{2}+b_{1}x_{2}\right)}{b_{i}^{2}+b_{1}x_{3}+x_{4}}\right]}^{2}$	4	[-5,5]
16	$F_{16}\left(x\right)=4x_{1}^{2}-2.1x_{1}^{4}+\frac{1}{3}x_{1}^{6}+x_{1}x_{2}-4x_{2}^{2}+4x_{2}^{4}$	2	[-5,5]
17	$F_{17}\left(x\right)={\left(x_{2}-\frac{5.1}{4\pi^{2}}x_{1}^{2}+\frac{5}{\pi }x_{1}-6\right)}^{2}+10\left(1-\frac{1}{8\pi }\right)\text{cos}x_{1}+10$	2	[-5,5]
18	$\begin{array}{c} F_{18}\left(x\right)=\left[1+{\left(x_{1}+x_{2}+1\right)}^{2}\left(19-14x_{1}+3x_{1}^{2}-14x_{2}+6x_{1}x_{2}+3x_{2}^{2}\right)\right]\\ \times \left[30+{\left(2x_{1}-3x_{2}\right)}^{2}\times \left(18-32x_{1}+12x_{1}^{2}+48x_{2}-36x_{1}x_{2}+27x_{2}^{2}\right)\right] \end{array}$	2	[-2,2]
19	$F_{19}\left(x\right)=-\overset{4}{\underset{i=1}{\sum }}c_{i}\text{exp}\left(-\overset{3}{\underset{j=1}{\sum }}a_{ij}{\left(x_{j}-p_{ij}\right)}^{2}\right)$	3	[1,3]
20	$F_{20}\left(x\right)=-\overset{4}{\underset{i=1}{\sum }}c_{i}\text{exp}\left(-\overset{6}{\underset{j=1}{\sum }}a_{ij}{\left(x_{j}-p_{ij}\right)}^{2}\right)$	6	[0,1]
21	$F_{21}\left(x\right)=-\overset{5}{\underset{i=1}{\sum }}{\left[\left(X-a_{i}\right){\left(X-a_{i}\right)}^{T}+c_{i}\right]}^{-1}$	4	[0,10]
22	$F_{22}\left(x\right)=-\overset{7}{\underset{i=1}{\sum }}{\left[\left(X-a_{i}\right){\left(X-a_{i}\right)}^{T}+c_{i}\right]}^{-1}$	4	[0,10]
23	$F_{23}\left(x\right)=-\overset{10}{\underset{i=1}{\sum }}{\left[\left(X-a_{i}\right){\left(X-a_{i}\right)}^{T}+c_{i}\right]}^{-1}$	4	[0,10]

### Result of benchmark functions during 1000 iterations

In this paper, the algorithms used for comparison include the traditional algorithms HHO [16], EO [56], TSA [57], GWO [17], SMA [18], and the improved algorithms SMA-CSA, ESMA [58], L-SHADE [59], CMA-ES [60], CSMA-1 [27], and CSMA-2 [27]. The software used for testing is MATLAB 2020b, and the hardware is a laptop computer with a 2.5GHz Intel(R) Core i5-7200U processor and 4GB memory. Each algorithm performs 20 independent operations to overcome randomness.

As shown in Table 4, in all experiments, the parameters of the comparative algorithms were the same as the recommended settings in their original works.

**Table 4:** Algorithms-specific parameter settings.

Algorithm	Parameter settings
SMA	N = 30, z = 0.03
GWO	N = 30, *a* = [2, 0]
TSA	*R* = 1, *P*_*min*_ = 1, *P*_*min*_ = 4
HHO	N = 30, beta = 1.5
ESMA	N = 30, z = 0.03
L-SHADE	N = 18D, H = 6, p = 0.11 and Arc rate = 2.6
CMA-ES	N = 4 + 3 log (D), μ = N/2
EO	*λ* = [0,1], *a*_1_ = 2, *a*_2_ = 1, GP = 0.5
CSMA	N = 30, z = 0.03

#### Comparison of benchmark function results between traditional algorithms

The results of the fitness test between SMA-CSA and the traditional algorithm are shown in Table 5, where the bolded experimental data are the best results for the selected data. Among them, for SMA-CSA, F1-F4 and F7 can achieve the best results and show excellent global convergence performance. For F5, SMA-CSA is second only to HHO; the performance is close. And F6 is worse than EO and HHO, but the result is still satisfactory, so it can be ranked third.

**Table 5:** Comparison results on benchmark functions with traditional algorithms.

Function		SMA-CSA	SMA	HHO	EO	GWO	TSA
F1	AVG	**0.000000E+00**	0.000000E+00	2.579614E-180	7.673746E-41	2.768645E-59	4.950208E-45
	STD	**0.000000E+00**	0.000000E+00	0.000000E+00	1.892294E-40	1.509283E-59	1.428727E-44
F2	AVG	**1.605563E-230**	3.341937E-228	8.411917E-97	8.200993E-23	1.152787E-33	5.709024E-29
	STD	**0.000000E+00**	0.000000E+00	1.969686E-96	1.081512E-22	1.710975E-33	7.080953E-29
F3	AVG	**0.000000E+00**	0.000000E+00	6.970295E-19	3.237446E-09	1.396782E-13	6.447270E-13
	STD	**0.000000E+00**	0.000000E+00	3.038281E-18	8.633206E-09	2.430992E-13	2.351925E-12
F4	AVG	**1.211135E-231**	7.540322E-193	1.902468E-89	4.443137E-10	1.383964E-13	3.072869E-03
	STD	**0.000000E+00**	0.000000E+00	8.228673E-89	5.696233E-10	2.132990E-13	4.628653E-03
F5	AVG	3.228899E-01	4.569646E-01	**1.354207E-03**	2.536489E+01	2.626128E+01	2.876574E+01
	STD	1.471756E-01	1.462580E-01	**1.769134E-03**	2.292356E-01	7.833016E-01	2.022235E-01
F6	AVG	3.896839E-04	1.326758E-03	3.430780E-05	**8.566418E-06**	5.088489E-01	4.104490E+00
	STD	3.738961E-04	8.219303E-04	3.653300E-05	**4.027177E-06**	2.953591E-01	8.608806E-01
F7	AVG	**2.153370E-05**	2.095878E-04	1.426068E-04	1.279508E-03	5.921268E-04	5.948308E-03
	STD	**2.492702E-05**	1.122627E-04	1.371302E-04	6.453354E-04	6.839246E-04	1.784391E-03
F8	AVG	**-1.256940E+04**	-1.256930E+04	-1.256920E+04	-8.809971E+03	-6.331787E+03	-6.234268E+03
	STD	**5.695252E-02**	1.360116E-01	2.401963E-01	5.559641E+02	9.978054E+02	6.639617E+02
F9	AVG	**0.000000E+00**	0.000000E+00	0.000000E+00	0.000000E+00	1.136868E-14	1.776554E+02
	STD	**0.000000E+00**	0.000000E+00	0.000000E+00	0.000000E+00	2.898457E-14	3.519182E+01
F10	AVG	**8.881784E-16**	8.881784E-16	8.881784E-16	8.348877E-15	1.652012E-14	1.570259E+00
	STD	**0.000000E+00**	0.000000E+00	0.000000E+00	1.548592E-15	2.842171E-15	1.215018E+00
F11	AVG	**0.000000E+00**	0.000000E+00	0.000000E+00	0.000000E+00	8.020254E-04	9.561414E-03
	STD	**0.000000E+00**	0.000000E+00	0.000000E+00	0.000000E+00	3.495948E-03	9.628837E-03
F12	AVG	1.942059E-04	1.265078E-03	6.626789E-07	**5.010153E-07**	3.474556E-02	8.613683E+00
	STD	2.875634E-04	7.434486E-04	6.667809E-07	**3.745496E-07**	1.650382E-02	1.689106E+00
F13	AVG	2.646654E-04	1.442929E-03	**5.258811E-05**	3.754184E-02	4.326672E-01	2.644201E+00
	STD	2.993505E-04	1.712270E-03	**3.652454E-05**	5.153452E-02	2.458081E-01	3.866198E-01
F14	AVG	**9.980038E-01**	9.980038E-01	1.047705E+00	9.980038E-01	1.978449E+00	5.254263E+00
	STD	2.626252E-13	2.329119E-14	2.166432E-01	**1.359740E-16**	2.576514E+00	4.380613E+00
F15	AVG	**2.835498E-04**	5.184759E-04	3.344348E-04	2.368814E-03	6.263507E-03	1.445361E-02
	STD	**1.367368E-04**	1.848918E-04	1.329292E-05	6.001443E-03	8.601985E-03	1.205828E-02
F16	AVG	**-1.031628E+00**	-1.031628E+00	-1.031628E+00	-1.031628E+00	-1.031628E+00	-1.030047E+00
	STD	3.543574E-11	1.179538E-09	3.914943E-11	**6.164821E-16**	2.767956E-08	6.893330E-03
F17	AVG	**3.978874E-01**	3.978874E-01	3.978880E-01	3.978874E-01	3.979093E-01	3.979168E-01
	STD	8.579928E-09	2.482701E-08	7.744872E-07	**0.000000E+00**	9.425006E-05	3.591852E-05
F18	AVG	**3.000000E+00**	3.000000E+00	3.000000E+00	3.000000E+00	3.000005E+00	2.869328E+01
	STD	7.166682E-12	7.251322E-13	1.815263E-07	**9.721058E-16**	1.029706E-05	3.676483E+01
F19	AVG	**-3.862782E+00**	-3.862782E+00	-3.862118E+00	-3.862388E+00	-3.861328E+00	-3.862369E+00
	STD	**9.993385E-08**	1.732100E-07	1.309203E-03	1.576306E-03	2.584704E-03	1.592744E-03
F20	AVG	-3.243908E+00	-3.221876E+00	-3.174149E+00	**-3.283054E+00**	-3.246269E+00	-3.268992E+00
	STD	5.611659E-02	**4.080885E-02**	7.312254E-02	6.096561E-02	7.175137E-02	5.762411E-02
F21	AVG	**-1.015315E+01**	-1.015308E+01	-5.807154E+00	-8.391108E+00	-1.015269E+01	-5.516240E+00
	STD	**5.116685E-05**	8.257390E-05	1.791615E+00	2.761756E+00	2.471429E-04	3.331850E+00
F22	AVG	**-1.040288E+01**	-1.040278E+01	-4.903478E+00	-8.340148E+00	-1.040186E+01	-6.948757E+00
	STD	**3.969636E-05**	7.966234E-05	1.669066E+00	2.839746E+00	4.540332E-03	3.146890E+00
F23	AVG	**-1.053633E+01**	-1.053621E+01	-5.947181E+00	-1.053641E+01	-1.053594E+01	-4.547859E+00
	STD	7.035456E-05	2.144832E-04	1.926396E+00	**6.782330E-09**	2.088163E-04	3.424523E+00

Note: The data in bold is the minimum value of all comparison algorithms.

Overall, SMA-CSA can obtain the global optimal solution in most cases and exhibits excellent convergence speed and local search capability.

For F9–F11 and F14, SMA-CSA can achieve optimal global convergence with the smallest standard deviation; for F12, it is worse than EO and HHO, can be ranked third; for F13, the global convergence performance is slightly inferior to HHO, ranking second and showing a better global search capability than the original SMA.

**Fig 3:**
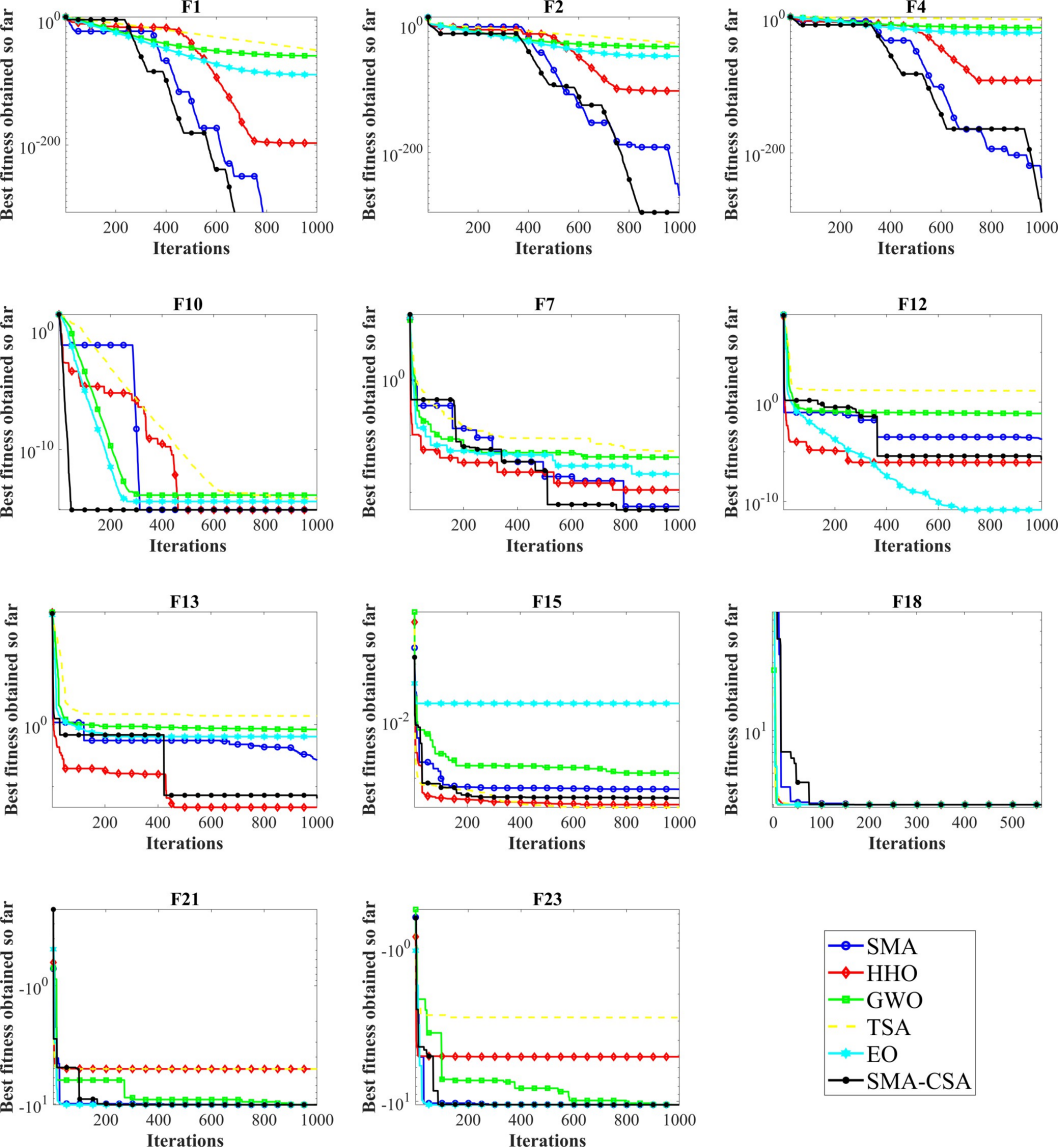
The convergence behavior of the comparative methods using CEC2013 problems. F1-F7 are single-modal benchmark functions; F8-F13 are multimodal benchmark functions; and F14-F23 are composite benchmark functions.

In addition, F15-F17 also can achieve optimal global solution, and have the smallest standard deviation and show excellent stability. For F21-F23, the average performance of SMA-CSA and SMA is equivalent, but SMA-CSA has the smallest standard deviation. Which also fully proves that SMA-CSA can significantly improve the equilibrium exploration ability and global search ability after adding the strategy of Cauchy mutation and simulated annealing. And Fig 3 shows the convergence curve of the traditional algorithm for some CEC2013 benchmark functions.

#### Comparison of benchmark function results between traditional algorithms with 60 dimensions

In order to highlight the high-dimensional optimization performance of SMA-CSA, this paper further tests 23 benchmark functions in 60 dimensions, and the results are shown in Table 6. SMA-CSA achieves the number one global value in most cases, such as F1-F5, F7, F8, F10, F11, F15, F16, and F18, and the variance stabilizes. However, SMA-CSA is inferior to HHO in F6 and F13. SMA-CSA still has excellent solution accuracy and global optimization capabilities, even in high-dimensional tests.

**Table 6:** Comparison of benchmark function results between traditional algorithms with 60 dimensions.

Functions		SMA-CSA	SMA	HHO	GWO	EO	TSA
F1	AVG	**0.0000E+00**	0.0000E+00	2.7476E-185	2.2659E-39	4.3300E-33	7.0864E-33
	STD	**0.0000E+00**	0.0000E+00	0.0000E+00	2.9668E-39	4.7600E-33	9.3181E-33
F2	AVG	**4.8216E-241**	4.4603E-172	5.8549E-99	1.3184E-23	1.8600E-18	2.0148E-21
	STD	**0.0000E+00**	0.0000E+00	8.0418E-99	6.2153E-24	2.4400E-18	1.2492E-21
F3	AVG	**0.0000E+00**	0.0000E+00	3.2064E-124	7.5613E-05	1.0452E-02	2.2634E+00
	STD	**0.0000E+00**	0.0000E+00	6.4127E-124	7.1374E-05	2.9743E-02	2.4793E+00
F4	AVG	**5.1209E-239**	3.3472E-201	3.9765E-97	9.3922E-09	8.5800E-06	8.0505E+00
	STD	**0.0000E+00**	0.0000E+00	7.3080E-97	4.9794E-09	2.8900E-05	4.0839E+00
F5	AVG	**3.8655E-02**	1.0757E+00	4.3459E-01	5.7049E+01	5.5587E+01	5.7829E+01
	STD	**2.8166E-02**	1.3489E+00	8.6011E-01	5.5307E-01	2.5392E-01	9.2755E-01
F6	AVG	1.7824E-02	3.5875E-02	**1.2866E-04**	3.2820E+00	3.4352E-01	6.9340E+00
	STD	1.2313E-02	1.6473E-02	**1.8968E-04**	9.8583E-01	2.9113E-01	4.1761E-01
F7	AVG	**2.5926E-05**	9.7826E-05	6.2958E-05	1.3287E-03	2.1980E-03	1.0142E-02
	STD	**1.5130E-05**	3.0592E-05	2.6627E-05	7.6195E-04	8.3800E-04	2.6394E-03
F8	AVG	**-2.5138E+04**	-2.5138E+04	-2.5134E+04	-1.0990E+04	-1.6752E+04	-1.0306E+04
	STD	**4.2073E-01**	5.6854E-01	8.4896E+00	6.3730E+02	7.9045E+02	6.6013E+02
F10	AVG	**8.8818E-16**	**8.8818E-16**	**8.8818E-16**	4.1389E-14	1.8800E-14	6.1292E-01
	STD	**0.0000E+00**	**0.0000E+00**	**0.0000E+00**	1.7405E-15	4.2300E-15	1.2258E+00
F11	AVG	**0.0000E+00**	**0.0000E+00**	**0.0000E+00**	**0.0000E+00**	8.6300E-04	8.5266E-03
	STD	**0.0000E+00**	**0.0000E+00**	**0.0000E+00**	**0.0000E+00**	3.8610E-03	7.0650E-03
F13	AVG	3.3577E-03	1.2580E-02	**2.8216E-05**	2.8281E+00	1.2131E+00	6.7418E+00
	STD	2.7792E-03	9.7061E-03	**1.8206E-05**	5.3487E-01	4.3662E-01	6.6120E-01
F15	AVG	**3.2199E-04**	5.6040E-04	3.3568E-04	7.3770E-03	4.3720E-03	1.2808E-02
	STD	**2.3822E-05**	3.3762E-04	2.8769E-05	8.7710E-03	8.2060E-03	9.4954E-03
F16	AVG	**-1.0316E+00**	-1.0316E+00	-1.0316E+00	-1.0316E+00	-1.0316E+00	-1.0316E+00
	STD	7.5976E-11	3.0601E-11	6.7319E-11	3.7888E-09	**1.9700E-16**	8.3828E-08
F18	AVG	**3.0000E+00**	3.0000E+00	3.0000E+00	3.0000E+00	3.0000E+00	3.0000E+00
	STD	3.7685E-14	8.1038E-13	1.0004E-09	7.8673E-06	**1.1100E-15**	5.1321E-05

Note: The data in bold is the minimum value of all comparison algorithms.

#### Comparison of benchmark function results between improved algorithms

In order to compare the comprehensive performance of SMA-CSA more fairly, other improved algorithms are compared in this paper, and the results are shown in Table 7. Among the 23 test functions, SMA-CSA can obtain the optimal global value in most cases, such as F1-F3, F7-F11, F14-F19, and F21-F23. Moreover, the variance tends to be stable. However, SMA-CSA is slightly inferior to L-SHADE in the F6, F12, and F13 test functions. Overall, even with the improved algorithm, SMA-CSA still has excellent solution accuracy and global optimization ability.

**Table 7:** Comparison of benchmark function results between improved algorithms.

function		SMA-CSA	SMA	ESMA	L-SHADE	CMA-ES	CSMA-1	CSMA-2
F1	AVG	**0.00E+00**	**0.00E+00**	**0.00E+00**	1.10E-27	8.24E-11	0.00E+00	0.00E+00
	STD	**0.00E+00**	**0.00E+00**	**0.00E+00**	1.77E-27	4.27E-11	0.00E+00	0.00E+00
F2	AVG	**1.61E-230**	2.85E-156	2.34E-184	2.95E-14	1.44E-05	5.11E-225	7.58E-210
	STD	**0.00E+00**	0.00E+00	0.00E+00	1.75E-14	4.58E-06	0.00E+00	0.00E+00
F3	AVG	**0.00E+00**	**0.00E+00**	**0.00E+00**	1.86E-13	2.29E+00	0.00E+00	0.00E+00
	STD	**0.00E+00**	0.00E+00	**0.00E+00**	4.51E-13	3.06E+00	0.00E+00	0.00E+00
F4	AVG	1.21E-231	2.30E-197	**6.64E-238**	2.75E-06	1.89E-04	2.15E-237	8.43E-236
	STD	**0.00E+00**	**0.00E+00**	**0.00E+00**	2.36E-06	6.40E-05	0.00E+00	0.00E+00
F5	AVG	3.23E-01	7.81E+00	3.76E+00	1.41E+01	6.50E+01	**1.48E-01**	1.78E-01
	STD	1.47E-01	6.37E-01	8.44E+00	8.50E-01	1.68E+02	1.19E-01	**9.57E-02**
F6	AVG	3.90E-04	6.13E-03	2.02E-03	**4.62E-27**	7.06E-11	9.59E-04	9.78E-04
	STD	3.74E-04	4.15E-04	8.36E-04	**2.20E-26**	3.67E-11	3.07E-04	6.18E-04
F7	AVG	**2.15E-05**	1.75E-04	1.50E-04	1.34E-03	5.23E-03	4.67E-05	5.16E-05
	STD	**2.49E-05**	7.12E-04	1.29E-04	3.64E-04	1.96E-03	3.32E-05	2.69E-05
F8	AVG	**-1.26E+04**	-1.26E+04	-1.26E+04	-3.26E+03	-inf	-1.26E+04	-1.26E+04
	STD	**5.70E-02**	3.36E-01	1.58E-01	4.15E+02	NaN	9.56E-02	6.65E-02
F9	AVG	**0.00E+00**	**0.00E+00**	**0.00E+00**	6.43E+00	1.62E+02	**0.00E+00**	**0.00E+00**
	STD	**0.00E+00**	**0.00E+00**	**0.00E+00**	1.46E+00	9.41E+00	**0.00E+00**	**0.00E+00**
F10	AVG	**8.88E-16**	8.88E-16	8.88E-16	4.20E-14	2.56E-06	8.88E-16	8.88E-16
	STD	**0.00E+00**	0.00E+00	0.00E+00	2.36E-14	6.98E-07	0.00E+00	0.00E+00
F11	AVG	**0.00E+00**	**0.00E+00**	**0.00E+00**	**0.00E+00**	4.57E-10	0.00E+00	0.00E+00
	STD	**0.00E+00**	**0.00E+00**	**0.00E+00**	**0.00E+00**	2.28E-10	0.00E+00	0.00E+00
F12	AVG	1.94E-04	2.99E-03	3.31E-03	**2.96E-16**	5.35E-12	3.19E-04	3.09E-04
	STD	2.88E-04	3.92E-03	4.86E-03	**1.54E-16**	2.83E-12	2.89E-04	2.30E-04
F13	AVG	2.65E-04	6.52E-03	3.60E-03	**9.67E-15**	5.27E-11	6.16E-04	4.41E-04
	STD	2.99E-04	6.83E-03	5.39E-03	**5.93E-15**	3.32E-11	3.22E-04	2.42E-04
F14	AVG	**9.98E-01**	**9.98E-01**	**9.98E-01**	8.63E+00	5.25E+00	9.98E-01	9.98E-02
	STD	2.63E-13	4.11E-13	4.11E-13	3.18E+00	3.57E+00	4.71E-14	1.26E-13
F15	AVG	**2.84E-04**	5.58E-04	5.61E-04	3.08E-04	3.79E-04	3.33E-04	3.45E-04
	STD	1.37E-04	2.50E-04	2.18E-04	**3.03E-19**	7.39E-05	3.99E-05	3.72E-05
F16	AVG	**-1.03E+00**	-1.03E+00	-1.03E+00	-1.03E+00	-1.03E+00	-1.03E+00	-1.03E+00
	STD	3.54E-11	2.34E-09	1.77E-09	**2.61E-16**	2.24E-16	1.76E-11	6.59E-11
F17	AVG	**3.98E-01**	3.98E-01	3.98E-01	7.79E+00	4.89E+00	3.98E-01	3.98E-01
	STD	**8.58E-09**	1.24E-07	1.15E-07	3.44E-01	6.50E-01	1.79E-08	1.37E-08
F18	AVG	**3.00E+00**	3.00E+00	3.00E+00	3.00E+00	3.00E+00	3.00E+00	3.00E+00
	STD	7.17E-12	1.41E-10	3.19E-11	**2.22E-15**	3.60E-15	9.55E-12	1.17E-11
F19	AVG	**-3.86E+00**	-3.86E+00	-3.86E+00	-3.86E+00	-3.86E+00	-3.86E+00	-3.86E+00
	STD	9.99E-08	3.21E-07	3.05E-06	3.14E-15	**3.14E-15**	1.55E-08	3.04E-08
F20	AVG	-3.24E+00	-3.25E+00	-3.25E+00	-3.29E+00	-3.26E+00	**-3.23E+00**	-3.24E+00
	STD	5.61E-02	6.00E-02	5.96E-02	5.09E-02	6.00E-02	**4.85E-02**	5.70E-02
F21	AVG	**-1.02E+01**	-1.02E+01	-1.02E+01	-5.06E+00	-8.30E+00	-1.02E+01	-1.02E+01
	STD	**5.12E-05**	2.52E-04	2.20E-04	5.63E-07	3.21E+00	8.16E-05	1.15E-04
F22	AVG	**-1.04E+01**	-1.04E+01	-1.04E+01	-5.09E+00	-1.04E+01	-1.04E+01	-1.04E+01
	STD	3.97E-05	2.73E-04	2.45E-04	1.25E-06	**8.97E-15**	8.53E-05	5.15E-05
F23	AVG	**-1.05E+01**	-1.05E+01	-1.05E+01	-5.26E+00	-1.02E+01	-1.05E+01	-1.05E+01
	STD	7.04E-05	3.23E-04	2.13E-04	6.32E-01	1.59E+00	7.01E-05	**6.47E-05**

Note: The data in bold is the minimum value of all comparison algorithms.

### Wilcoxon rank sum test and Friedman’s ranking test

In order to compare the performance differences between the algorithms more clearly, we adopted the literature recommendation reference [61] and performed the nonparametric Wilcoxon rank sum test on the experiment at the p = 0.05 significant level [62]. The experimental results used to verify SMA-CSA, SMA, and other comparison algorithms are statistically different. Finally, the Friedman ranking test is performed on all test results, aiming to show the algorithmic computing performance gap more intuitively. The results of the Wilcoxon rank-sum test are shown in Table 8.

**Table 8:** Comparison results on Wilcoxon rank sum test with algorithms.

Function	SMA	HHO	EO	GWO	TSA
F1	NaN	8.01E-09	8.01E-09	8.01E-09	8.01E-09
F2	2.97E-01	6.70E-08	6.70E-08	6.70E-08	6.70E-08
F3	NaN	8.01E-09	8.01E-09	8.01E-09	8.01E-09
F4	9.73E-05	6.76E-08	6.76E-08	6.76E-08	6.76E-08
F5	8.40E-03	6.80E-08	6.80E-08	6.80E-08	6.78E-08
F6	7.41E-05	2.96E-07	6.80E-08	6.80E-08	6.80E-08
F7	3.66E-07	1.41E-05	6.80E-08	2.21E-07	6.80E-08
F8	8.17E-02	3.40E-03	4.94E-08	4.94E-08	4.94E-08
F9	NaN	NaN	NaN	8.06E-02	7.96E-09
F10	NaN	NaN	7.43E-10	2.04E-09	7.99E-09
F11	NaN	NaN	NaN	3.42E-01	9.43E-06
F12	5.25E-05	2.69E-06	1.04E-06	6.77E-08	6.77E-08
F13	9.74E-06	1.14E-02	8.10E-02	6.80E-08	6.80E-08
F14	1.97E-01	1.59E-05	7.95E-09	6.46E-08	6.07E-08
F15	1.43E-04	2.75E-02	3.94E-01	1.33E-01	8.59E-06
F16	1.02E-01	5.34E-01	8.01E-09	6.80E-08	1.61E-04
F17	9.68E-01	9.79E-03	8.01E-09	8.36E-04	1.17E-05
F18	1.94E-02	5.51E-03	1.58E-03	6.36E-08	6.44E-08
F19	5.61E-01	2.78E-07	6.22E-04	1.43E-07	1.20E-06
F20	1.07E-01	3.33E-03	6.37E-05	4.73E-01	7.35E-01
F21	9.05E-03	6.80E-08	4.89E-01	3.50E-06	6.79E-08
F22	2.59E-05	6.79E-08	1.01E-01	3.29E-05	6.79E-08
F23	7.11E-03	1.14E-07	3.53E-07	6.92E-07	6.80E-08

Note: Wilcoxon rank-sum test at the p = 0.05 significant level.

NaN: represents the same test value.

The test results show that, in most cases, there are performance differences between SMA-CSA and other algorithms. There are five benchmark functions with the same results for the test results of the SMA-CSA and SMA algorithms, and the ideal and optimal results are achieved. The null hypothesis cannot be accepted for F4, F5, F6, F7, F12, F13, F15, F18, F21, F22, and F23 benchmark function test results at the p = 0.05 significant level. That is, the global performance of SMA-CSA and SMA is significantly different. However, for F2, F8, F14, F16, F17, F19, and F20 functions, at the p = 0.05 significant level, the null hypothesis is not rejected, so the performance of SMA-CSA is similar to the original SMA.

In addition, only discussing the results of the Wilcoxon test lacks intuitive feelings, so the Friedman ranking test is added for comprehensive ranking [63]. In addition, separate rankings are performed according to the types of different benchmark functions, and finally, the mean ranking of all benchmark functions is given. As shown in Tables 9–11, for the single-peak and multi-peak test functions F1-F13, the mean ranking of SMA-CSA is 1.8462, ranking it first ahead of other algorithms, and its performance is 27.27% higher than that of SMA. HHO and SMA are in SMA-SA, followed in second and third place, respectively. It can be seen that SMA-CSA has better optimization performance for unimodal and multimodal benchmark functions.

**Table 9:** Comparison results on Friedman’s ranking test with traditional algorithms.

Test problems	Metric	Optimization algorithms					
		SMA-CSA	SMA	HHO	EO	GWO	TSA
**Scalable test problems**	**Mean rank**	1.8462	2.5385	2.3077	3.9231	4.6154	5.7692
**F1-F13**	**Rank**	1	3	2	4	5	6
**Non-scalable test problems**	**Mean rank**	2	2.4	4.1	2.9	4.3	5.3
**F14-F23**	**Rank**	1	2	4	3	5	6
**Total test problems**	**Total Mean rank**	1.913	2.4783	3.087	3.4783	4.4783	5.5652
**F1-F23**	**Total Rank**	1	2	3	4	5	6

**Table 10:** Comparison results on Friedman’s ranking test with traditional algorithms with 60 dimensions.

Test problems	Metric	Optimization algorithms					
		SMA-CSA	SMA	HHO	GWO	EO	TSA
**Scalable test problems**	**Total Mean rank**	1.82	2.54	2.39	4.25	4.43	5.57
**F1-F18**	**Total Rank**	1	3	2	4	5	6

**Table 11:** Comparison results on Friedman’s ranking test with advanced algorithms.

Test problems	Metric	Optimization algorithms						
		SMA-CSA	SMA	ESMA	L-SHADE	CMA-ES	CSMA-1	CSMA-2
**Scalable test problems**	**Total Mean rank**	3.1304	4.2174	3.9130	4.8478	5.0652	3.4130	3.4130
**F1-F23**	**Total Rank**	1	5	4	6	7	2	3

For the composite benchmark function F14-F23, the mean ranking of SMA-CSA is 2.00, ranking it first, which is significantly better than SMA and other algorithms, and the performance is improved by 16.67% compared with SMA. It shows that for the composite benchmark function, the global optimization performance of the original SMA can be improved by introducing a mixed strategy of annealing and Cauchy mutation. And the results in Table 10 show that in the benchmark function measurement under 60 dimensions, the performance improvement of SMA-CSA is still obvious. From the perspective of average ranking, compared with the original SMA, the optimization performance is up 28.35%.

Looking at the 23 benchmark functions in general, compared with the traditional algorithm, the mean ranking of SMA-CSA is 1.913, which is better than other algorithms and ranks first. Compared with the original SMA, the performance is improved by 22.81%; the mean ranking of the SMA is 2.4783, ranking it in second place.

Secondly, compared with the advanced algorithms, the mean ranking of SMA-CSA is 3.1304, ranking first, which is 25.77% higher than that of the original SMA. Moreover, the mean ranking of CSMA-1 is 3.4130, ranking it second, and the performance of SMA-CSA is slightly better than that of CSMA-1, an increase of 8.3%. And the performance of SMA-CSA is 20.00% higher than that of ESMA. The test results once again prove the effectiveness of the optimization performance improvement introduced by introducing the annealing and Cauchy mutation hybrid strategy.

### Impact analyses of SA and CM

**Fig 4:**
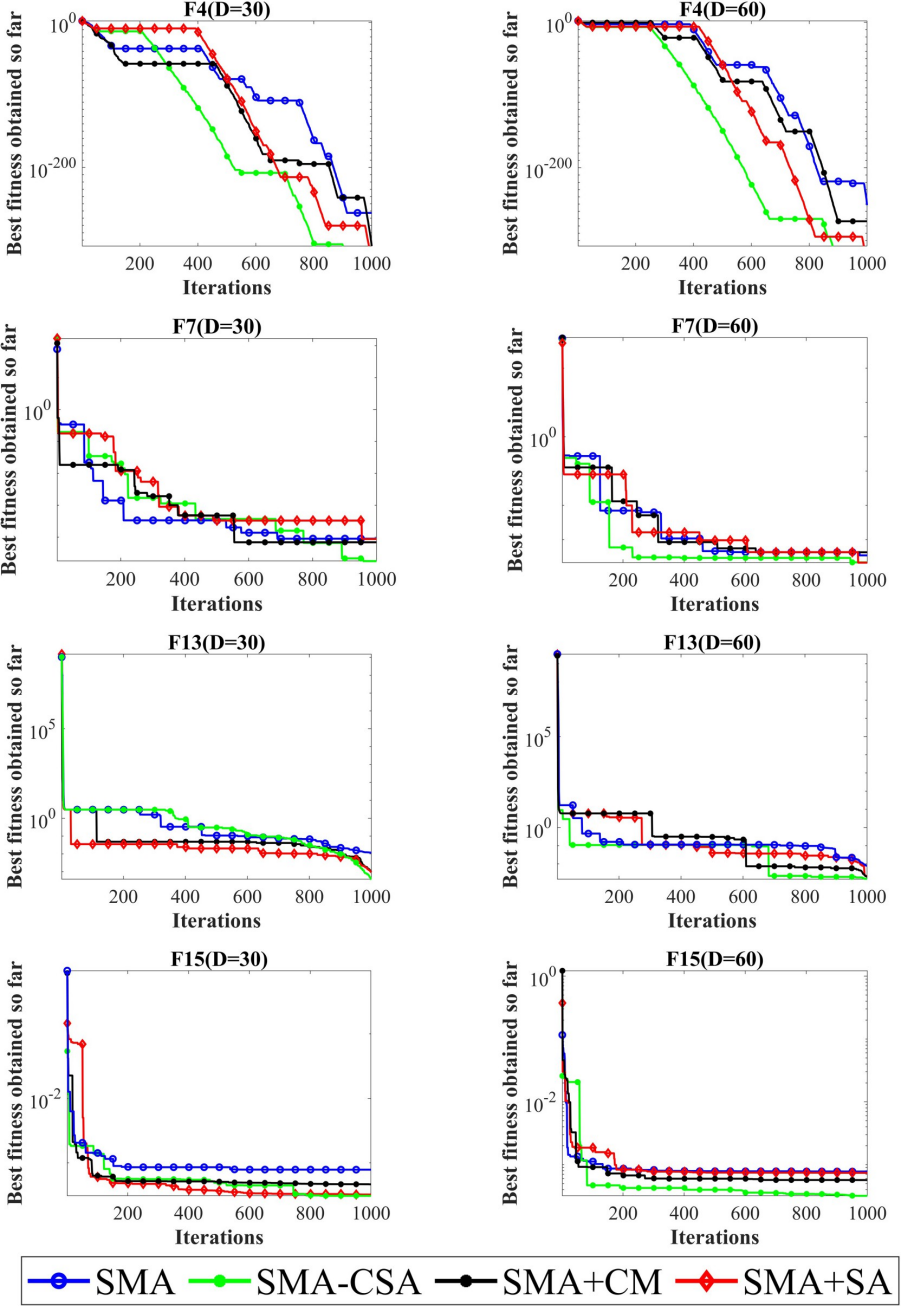
Fitness values obtained by SMA, SMA+SA, SMA+CM and SMA-CSA.

In this experiment, the impact of the Cauchy mutation search strategy and simulated annealing operator on the performance of the SMA algorithm is analyzed. The results of these analyses are shown in Fig 4. Four functions (F4, F7, F13, and F15) are selected, and each function is from CEC 2013. In order to analyze the impact of different strategies on SMA, four algorithms SMA, SMA+SA, SMA+CM, and SMA-CSA were developed and compared. Fig 4 shows the slime mold algorithm in different dimensions of CEC 2013 (D = 30; D = 60) and the best fitness of each iteration of the partially selected function. As the curves shown in this figure show, for the functions F4, F7, F13, and F15, the solutions obtained by SMA+SA and SMA+CM are all better than those obtained by SMA, which shows the influence of SA and CM in development and exploration. There is more benefit to using both SA and CM than the solutions obtained by SMA + SA and SMA + CM because the solutions obtained by SMA-CSA are always better than those obtained by SMA + SA and SMA + CM. In addition, for functions F4, F7, F13, and F15, the solution of SMA+SA is better than that of SMA+CM, indicating that the SA strategy contributes more to improving the optimization accuracy.

### Experiments on CVRP

#### Problem description

A mathematical model can describe the capacitated vehicle routing problem (CVRP): A distribution center O provides logistics services for N customers, where the number of logistics vehicles is M and the maximum load capacity of each vehicle is Q.


$$M=\left\{k\right\},\;k=1,2,\cdots ,m$$

The logistics vehicles must provide logistics services for customers from the distribution center and return to the center after completing their tasks.


$$V=\left\{i\right\},\;i=1,2,\cdots ,n$$

All logistics vehicles must serve customers at all nodes within their capacity Q limits, with no omissions.


$$Q_{k}\leq Q,\;k\in M;\;Q\geq max\left\{q_{i},which\;i\in V\right\}$$

The final capacity of the vehicle is linked to the number of goods to be delivered at each node and the number of nodes (customers) to be served by the vehicle on the route.

Building the CVRP mathematical model:

$$m=\left[\frac{\sum q_{1}}{Q}\right]$$ (13)

$$f_{1}=min\overset{K}{\underset{k=1}{\sum }}\overset{V}{\underset{i=0}{\sum }}\overset{V}{\underset{j=0}{\sum }}c_{ij}x_{ijk}$$

$$f_{2}=\left(max\left\{\overset{K}{\underset{k=1}{\sum }}\overset{V}{\underset{i=0}{\sum }}\overset{V}{\underset{j=0}{\sum }}c_{ij}y_{ik}\right\}\right)$$ (14)

$$fitness=f=w_{1}\cdot f_{1}+w_{2}\cdot m\cdot f_{2}$$ (15)

$$\overset{n}{\underset{j=0}{\sum }}x_{ijk}=y_{ki};\overset{n}{\underset{i=0}{\sum }}x_{ijk}=y_{kj}$$ (16)

$$\overset{n}{\underset{i=1}{\sum }}q_{i}y_{ki}\leq Q,k\in M$$ (17)

$$\overset{m}{\underset{k=1}{\sum }}y_{k0}=m$$ (18)

$$\underset{k\in M}{\sum }y_{ik}=1,i\in V$$ (19)

$$\underset{i,j\in S\times S}{\sum }x_{ijk}\leq \left|S\right|-1,S\in \left\{1,2,\cdots ,n\right\}$$ (20)

$$x_{ijk}\in \left[0,1\right],y_{ik}\in \left[0,1\right]$$ (21)

$$F=\min\left\{f_{1}\right\}$$ (22)

Where m is the number of vehicles; *q*_*i*_ represents the quantity demanded by the customer, *i* ∈ *V*; (*q*_0_ represent the warehouse); *c*_*ij*_ represents the distance from customer *i* to customer *j*; *c*_0*j*_ indicates the distance from the warehouse to customer *j*; *c*_*i*0_ represents the distance from customer *i* to the warehouse; If vehicle *k* visits client *i*, let *y*_*ik*_ = 1, otherwise *y*_*ik*_ = 0; If vehicle *k* continues to visit customer *j* after visiting customer *i*, let *x*_*ijk*_ = 1, otherwise *x*_*ijk*_ = 0.

And *f*_1_ is the desired objective function, that is, the shortest total distribution path length; *f*_2_ is the longest path for a single vehicle; *f* is the individual fitness; the constraint (4.4) indicates that there is one and only one vehicle from one node to another; (Eq 17) indicates that the transportation capacity of a vehicle must not exceed its own maximum carrying capacity; Eq (18) indicates that the starting point of a vehicle and the ending point of a vehicle are warehouses; Eq (19) ensures that each vehicle is visited (except warehouses); Eq (20) is used to eliminate subloops; Eq (21) is a range of parameter values; and finally Eq (22) is the problem of the minimum path we need to solve.

#### Hybrid SMA-CSA for CVRP

**Fig 5:**
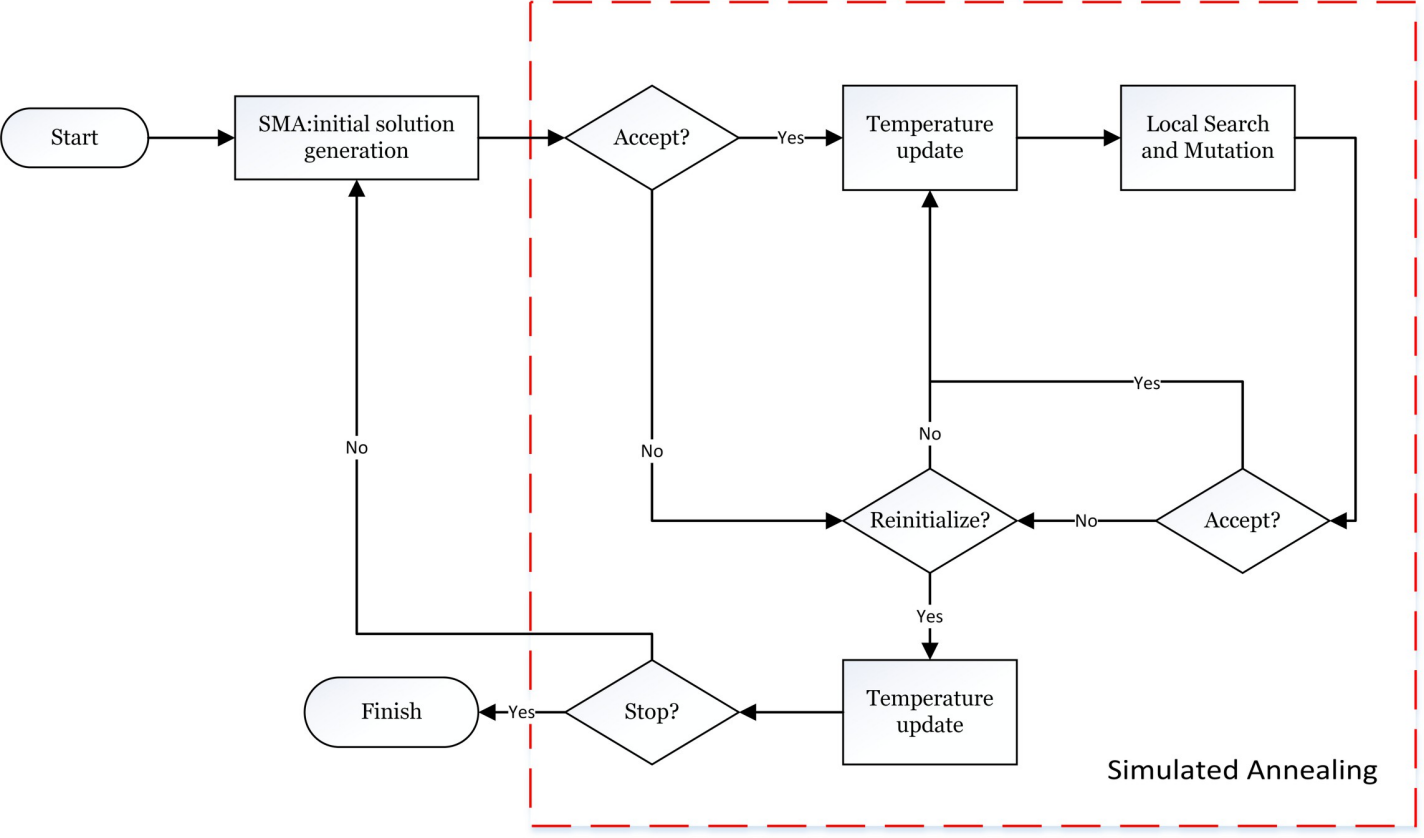
Flowchart of SMA-CSA for CVRP.

The main content of this section is to design the application idea of SMA-CSA for the capacitated vehicle routing problem, and the algorithm structure and the steps are shown in Fig 5. Two standard benchmark datasets of CVRP are selected reference [64] and [65], and the results are compared with other algorithms to analyze the advantages and disadvantages of different algorithms for solving the problem. The first of these data sets has the number of instance nodes between 50–199; the second is distributed between 200–483. The distances between customer and customer nodes and between customer and warehouse nodes are measured using Euclidean distance.

*1) Initialization*. First, the algorithm initialization parameters are defined, including population size, maximum iterations, and crossover (C-R), and then the algorithm randomly generates the initial population of slime mould.

The formation of the optimal solution (*X*_*i*_) for each individual slime mould is based on the following steps:

(Step1). Create a path that uses the warehouse as the starting point (zero points);
(Step2). select a customer from the customer list in a random non-replacement form;
(Step3). Add the selected customer to the route;
(Step4). Determine whether the total demand of all customers on the path is less than the vehicle capacity;
(Step5). Repeat step2 and step3, otherwise add the warehouse to the route;
(Step6). Add a route;
(Step7). Repeat steps 1–6 until all customers have been traversed;
So far, each individual’s solution (*X*_*i*_) has been calculated, sorting the solution *X*_*i*_ of all slime mould, and calculating the weight of the corresponding solution, finally updating the optimal solution.

*2) Crossover*. When using the SMA-CSA algorithm to solve CVRP, introduce crossover (C-R) in the process of position updating to improve the quality of the solution. In the crossover, an individual is selected first, and then new positions P1 and P2 are randomly generated between the solution set, and the positions of the newly generated solutions are positioned between P1 and P2.

*3) Local search*. In order to expand the range of the solutions, three local search strategies are used, including point-swap, 2-opt, and 3-opt.

**Fig 6:**
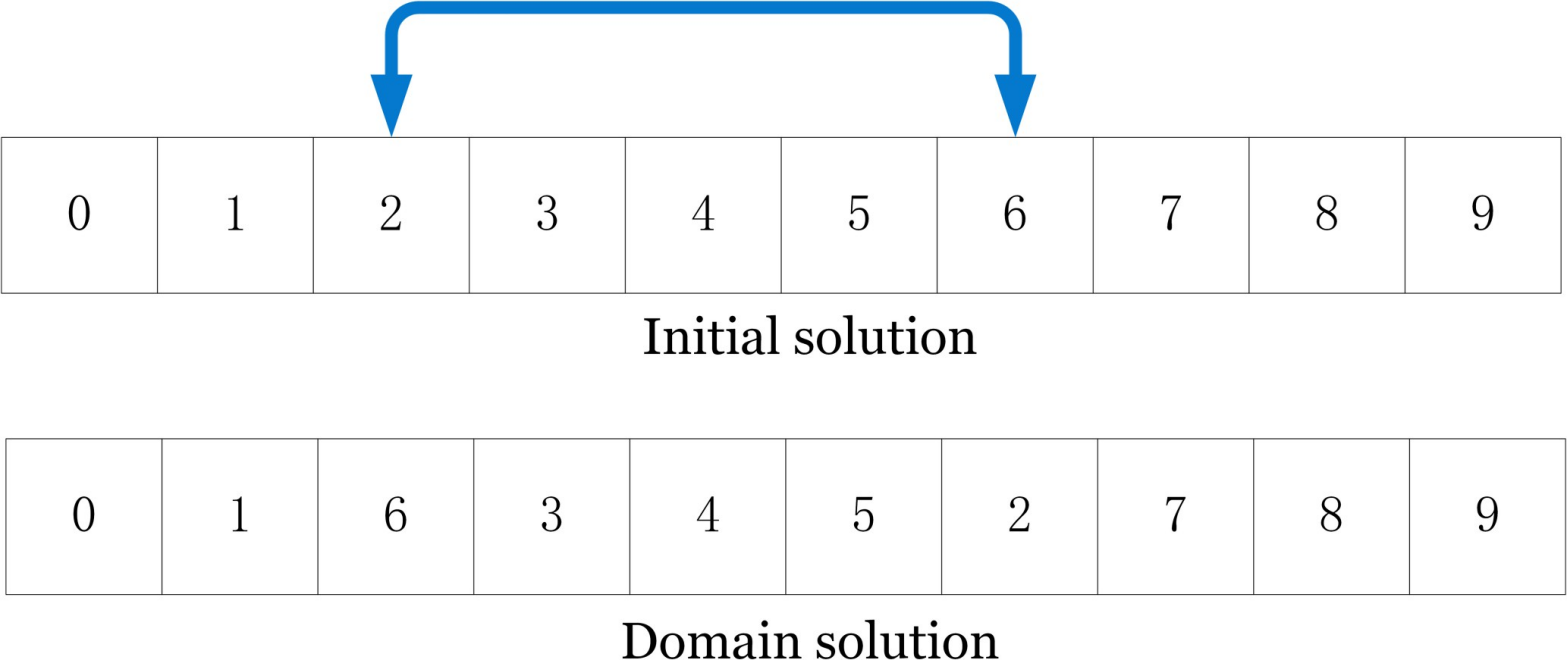
Swap example diagram.

***(1) Point swap strategy*.** As shown in Fig 6, in the current solution, two customers at different locations are randomly selected and swapped to generate a new domain solution.

**Fig 7:**
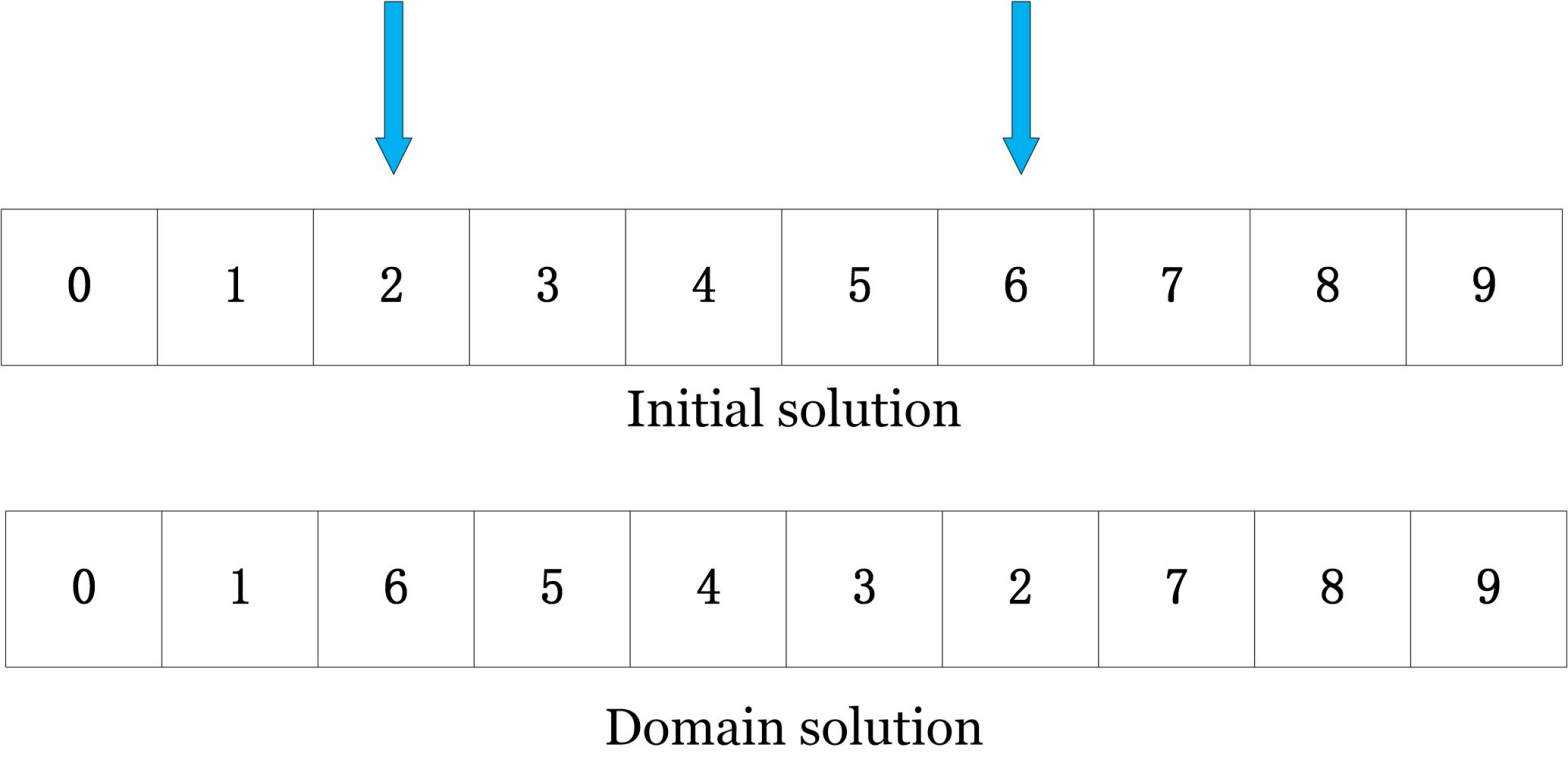
2-opt example diagram.

***(2) 2-opt search*.** As shown in Fig 7, similar to the point swap strategy, randomly generate two customer points at different locations, flip the path between the two customer points, and add their numbers to the new path; the path number before and after the two customer points remains unchanged.

**Fig 8:**
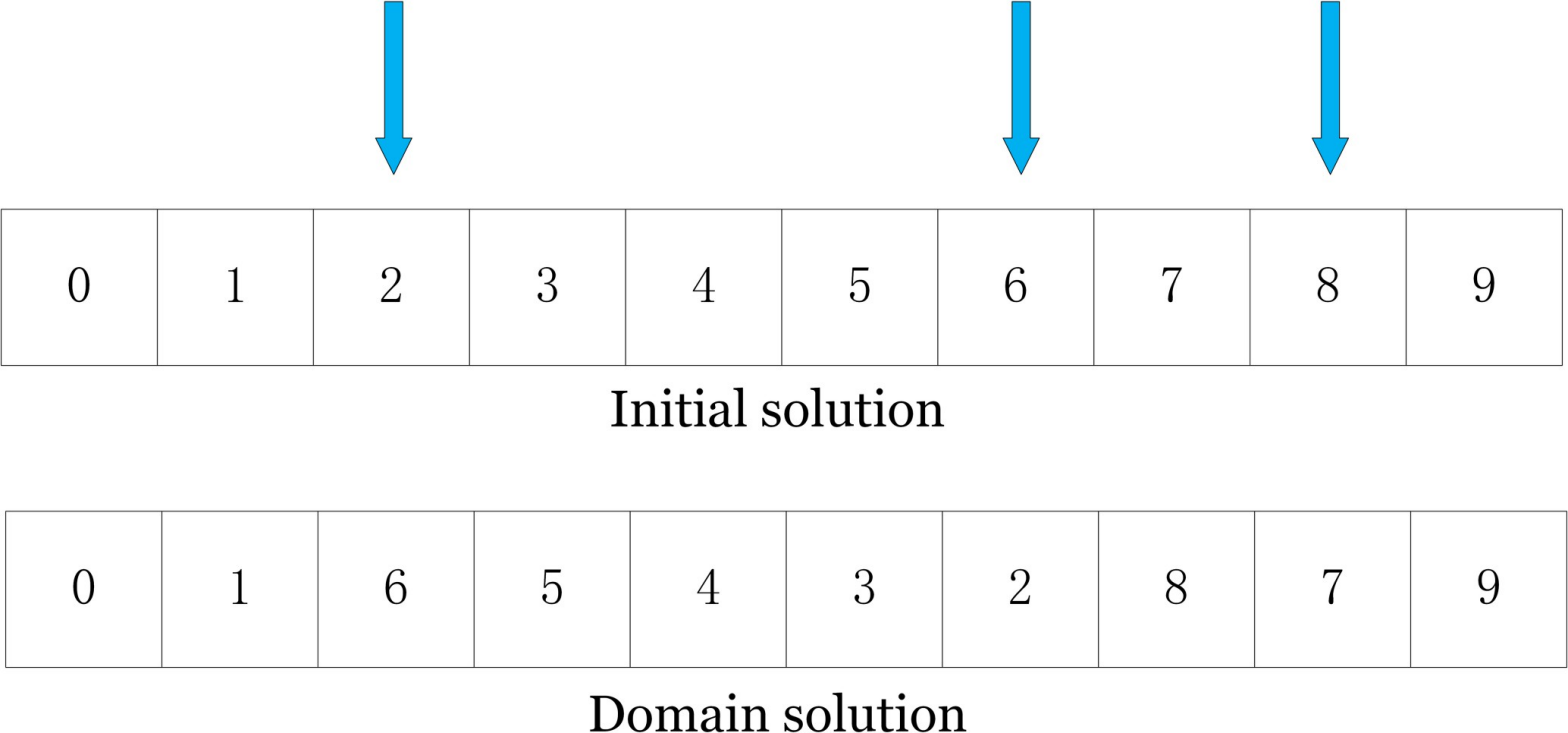
3-opt example diagram.

***(3) 3-opt search*.** As shown in Fig 8, Similarly, the 3-opt search strategy is similar to the 2-opt search strategy, in which three location client points are randomly selected for the path and swapped and flipped in turn.

*4) Mutation*. In order to ensure the global search ability of the algorithm, this paper uses three mutation operators with different selection probabilities to improve the diversity of the solutions. The first mutation randomly selects two customers in the CVRP solution with two paths and swaps the two customers in each of the two paths. The second mutation randomly selects two routes in the CVRP solution and swaps the customers within the two paths to form a new path. The third mutation is the 2-h-opt mutation and is one of the most effective mutations for complex problems [66].

First, the multi-vehicle path is converted into a single-vehicle path problem using the predefined probability M-R. Then, the 2-h-opt operator is used to find the twisted connections in the original path and reopen them to generate a new multi-vehicle problem.

#### Result of CVRP

This section discusses the results of solving CVRP using SMA-CSA and analyzes its performance. Two standard benchmark datasets of CVRP are selected, and the results are compared with other algorithms to analyze the advantages and disadvantages of different algorithms for solving the problem. The algorithms used for comparison include SMA-CSA, ISOS, EACO [67], LNS-ACO [68], ILS–RVND [69, 70], GRELS [71], AGES [72], and HGPSO [73].

The test results of the selected cases are shown in Tables 13 and 14. In order to make the final results converge to the optimal value, two stopping criteria are designed in this paper. The first is that the result of the cases is equal to the optimal result BKS (best known), and the second is that the maximum iteration value of the algorithm is reached.

Secondly, the parameters related to SMA-CSA and the comparison algorithm are set, as shown in Table 12. Each case is executed ten times, respectively, and the optimal value is taken for comparison. The average gap between the actual value and the optimal value of the cases is then calculated.

**Table 12:** Algorithms-specific parameter settings.

Algorithm	Parameter settings
SMA-CSA	MI = 2000, n = 30, C-R = 0.95, M-R = 0.1, Pop-size = 30, z = 0.03
ILS-RVND	MI = 2000, a = 0.05, b = 0.005, N = 150, A = 11
ISOS	MI = 1000, eco_size = 25, 50, 75, T0 = 15, 25,35, pf = 0.7, 0.8, 0.9
EACO	MI = 2000, r1 = 0.5, r2 = 0.3, r3 = 0.2, α = 1, β = 1, p = 6
LNS-ACO	MI = 5000, r1 = 0.5, r2 = 0.3, r3 = 0.2, α = 1, β = 1, q = 4≤ randi() ≤min(100,0.4n), p = 6

In Table 13, only in the C1, C12, and C14 cases do the solutions of all variants obtain results consistent with the optimal values. In the C3, C6, and C8 cases, all the algorithms obtain results consistent with BKS, except for ILS-RVND. In C2 and C7, SMA-CSA, EACO, and LNS-ACO outperform ISOS and ILS-RVND. In terms of average solutions, among all algorithms, the average gap of SMA-CSA is significantly smaller than that of other algorithms, showing a better and more stable global search capability than other algorithms.

**Table 13:** Comparison results on Christofides’s benchmark datasets of CVRP with advanced algorithms.

Instance		BKS (Best known)	ILS–RVND	ISOS	EACO	LNS-ACO	SMA-CSA
Name	n						
C1	50	524.61	524.61	524.61	524.61	524.61	524.61
C2	75	835.26	839.75	835.74	835.26	835.26	835.26
C3	100	826.14	827.63	826.14	826.14	826.14	826.14
C4	150	1028.42	1030.65	1028.42	1041.83	1046.9	1028.42
C5	199	1291.29	1306.63	1305.49	1338.48	1341.4	1302.103
C6	50	555.43	557.56	555.43	555.43	555.43	555.43
C7	75	909.68	914.56	914.68	909.68	909.68	909.68
C8	100	865.94	869.61	865.94	865.94	865.94	865.94
C9	150	1162.55	1170.76	1162.55	1168.81	1164.93	1162.78
C10	199	1395.85	1405.83	1417.9	1413.69	1419.7	1412.44
C11	120	1042.11	1042.11	1042.11	1045.5	1042.11	1040.36
C12	100	819.56	819.56	819.56	819.56	819.56	819.56
C13	120	1541.14	1547.39	1541.14	1554.93	1547.1	1542.21
C14	100	866.37	866.37	866.53	866.37	866.38	866.37
Avg.Gap			0.45%	0.24%	0.57%	0.57%	0.14%

It can be seen from Table 14 that only in the GWKC5 example does SMA-CSA achieve the best results. In the cases of GWKC3, GWKC6, GWKC9, and GWKC17, actual results that are close to optimal results were obtained. In addition, it can be seen from the table that it seems that when the number of nodes n is large, the actual result of SMA-CSA is not particularly ideal; when n is small, it is close to the optimal result. In terms of average solution, among all algorithms, the average gap size of SMA-CSA is second only to AGES, and its performance is good.

**Table 14:** Comparison results on Golden’s benchmark datasets of CVRP with advanced algorithms.

Instance		BKS (Best known)	GRELS	AGES	EACO	HGPSO	SMA-CSA
Name	n						
GWKC1	240	5623.47	5644.52	5627.54	5627.54	5670.38	5627.54
GWKC2	320	8404.61	8447.92	8447.92	8496.68	8459.73	8426.73
GWKC3	400	11036.20	11036.22	11036.22	11175.30	11101.12	11036.22
GWKC4	480	13590.00	13624.52	13624.52	14244.60	13698.17	13634.01
GWKC5	200	6460.98	6460.98	6460.98	6512.27	6460.98	6460.98
GWKC6	280	8412.80	8412.9	8412.88	8412.80	8470.64	8412.82
GWKC7	360	10102.70	10195.59	10195.56	10420.80	10215.14	10201.3
GWKC8	440	11635.30	11643.9	11663.55	12233.80	11750.38	11683.17
GWKC9	255	579.71	586.23	583.39	583.39	586.87	579.8
GWKC10	323	735.66	744.36	741.56	766.55	746.56	744.36
GWKC11	399	912.03	922.4	918.45	946.61	925.52	922.73
GWKC12	483	1101.50	1116.12	1107.19	1152.68	1114.31	1123.63
GWKC13	252	857.19	862.32	859.11	875.71	865.19	859.11
GWKC14	320	1080.55	1089.35	1081.31	1106.41	1089.21	1080.9
GWKC15	396	1337.87	1352.39	1345.23	1373.40	1355.28	1352.39
GWKC16	480	1611.56	1634.27	1622.69	1682.88	1632.21	1629.17
GWKC17	240	707.76	708.85	707.79	707.79	712.18	707.79
GWKC18	300	995.13	1002.15	998.73	1024.51	1006.31	995.82
GWKC19	360	1365.60	1371.67	1366.86	1399.95	1373.24	1371.31
GWKC20	420	1817.59	1830.98	1820.09	1821.15	1831.17	1839.68
Avg.Gap			0.64%	0.34%	2.35%	0.92%	0.53%

**Fig 9:**
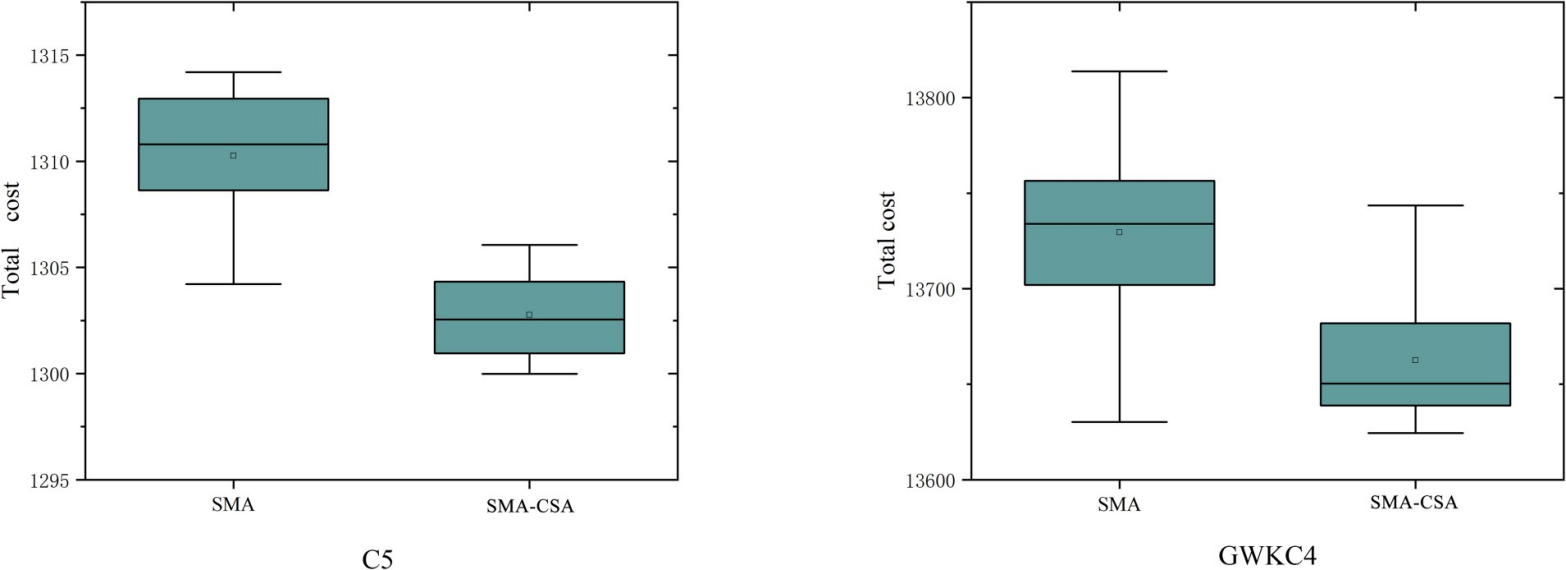
Comparison of results between SMA and SMA-CSA in C5 and GWKC4 examples.

In order to observe the results of SMA and SMA-CSA in multiple operations more intuitively, Fig 9 draws the boxplots of the C5 and GWKC4 examples. In 10 operations, SMA-CSA has better optimization results and a more robust mean than other algorithms.

**Fig 10:**
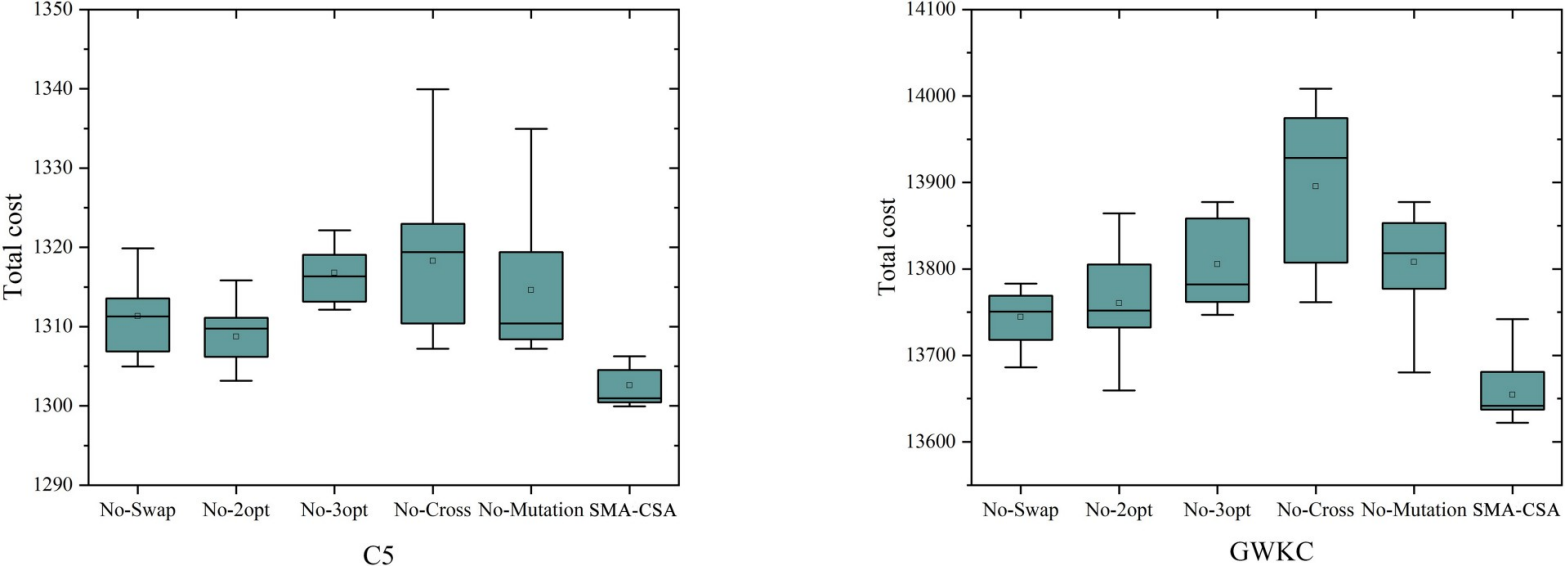
Results when one of the local search or mutation strategies is unused on C5 and GWKC4.

In order to verify the effect of the article search strategy, the article calculates the results when SMA-CSA does not apply a local search strategy or crossover mutation strategy. It can be seen from Fig 10 that when the crossover and mutation operations are not applied, the difference in the algorithm optimization results is the most obvious; in the local search strategy, when 2-opt is not used, the algorithm optimization results are greatly affected.

## Conclusion

Metaheuristic algorithms show that they are able to arrive at optimal solution sets for global optimization and discrete problems in a reasonable amount of time. Some of the most representative metaheuristic algorithms include: Moth Search (MS) [74], Earthworm Optimization Algorithm (EWA) [75], Harris Hawk Optimization (HHO) [16], Slime Mold Algorithm (SMA) [18], Runge Kutta Optimizer (RUN) [76], Crowd Predation Algorithm (CPA) [77], and so on. However, the complexity of different algorithms is inconsistent, the difficulty of discrete coding is different, and most of them lack group diversity and good search strategies, which leads to premature convergence of the local optimum, so introducing a suitable search mutation strategy is an improvement idea for most algorithms.

It was the main inspiration of this study to propose an improved slime mold algorithm named SMA-CSA based on Cauchy mutation and simulated annealing hybrid strategy optimization and then recoded to solve two CVRP data sets. To address the SMA’s shortcomings, the SMA-CSA implemented the Cauchy mutation operator, random perturbation, and the Metropolis sampling criterion acceptance strategy.

The proposed SMA-CSA algorithm is evaluated on global optimization problems using CEC 2013, and its performance is compared to that of SMA and other algorithms such as HHO, SMA, EO, TSA, GWO, ESMA, and L-SHADE, CMA-ES, and CSMA.

Different mathematical and structural examples are considered for evaluating the performance of the new algorithm. The good results of SMA-CSA in benchmark functions (F1–F7) show the performance of the SMA-CSA algorithm in terms of exploitation and local optimum avoidance. Moreover, the algorithm is applied to the capacitated vehicle routing problem (CVRP) and compared to other algorithms such as ISOS, EACO [67], LNS-ACO [68], ILS–RVND [69, 70], GRELS [71], AGES [72], and HGPSO [73], the results prove SMA-CSA has better optimization results and a more robust mean than other algorithms.

Although the research results of the article confirm the feasibility of annealing and Cauchy mutation mixed strategy coordination optimization, there are still some limitations in the overall research. For example, although the convergence speed and global accuracy of the improved SMA are significantly improved, the calculation time is increased by 57% on average compared with the SMA due to the introduction of the annealing process, which requires longer computation time. Secondly, the improved algorithm does not further calculate the test results of different benchmark functions in more dimensions and does not verify the impact of dimensional changes on the algorithm simulation results. Thirdly, the amount of data analysis in the CVRP example used in this article is relatively small, and there is a lack of solution analysis for large-scale node data, so the conclusion about algorithm performance has limitations. Finally, the use of CVRP problems to test the discrete solution performance of the SMA-SA algorithm is still not convincing, and the ability of SMA-SA to solve discrete problems should be further tested in more practical optimization problems (like scheduling, image processing, medical applications, etc.).

In addition, future researchers can further optimize the slime mold algorithm from other perspectives, such as by introducing a reverse learning mechanism while adaptively controlling algorithm parameters, which may lead to better results. In addition, applying the SMA-SA algorithm to VRPTD, VRPTW, GVRP, and other similar vehicle routing problems and real-case applications to further verify the performance of the algorithm is an effective research idea.

## Supporting information

S1 Algorithm Pseudo-code for Algorithm 1. (TIF)

S2 Algorithm Pseudo-code for Algorithm 2. (TIF)

S1 Appendix (DOCX)
