# Supplementary figures and images for: Improved slime mould algorithm based on hybrid strategy optimization of Cauchy mutation and simulated annealing
Source: PLoS One. 2023 Jan 25;18(1):e0280512. doi: 10.1371/journal.pone.0280512 (PMC9876378; doi:10.1371/journal.pone.0280512)

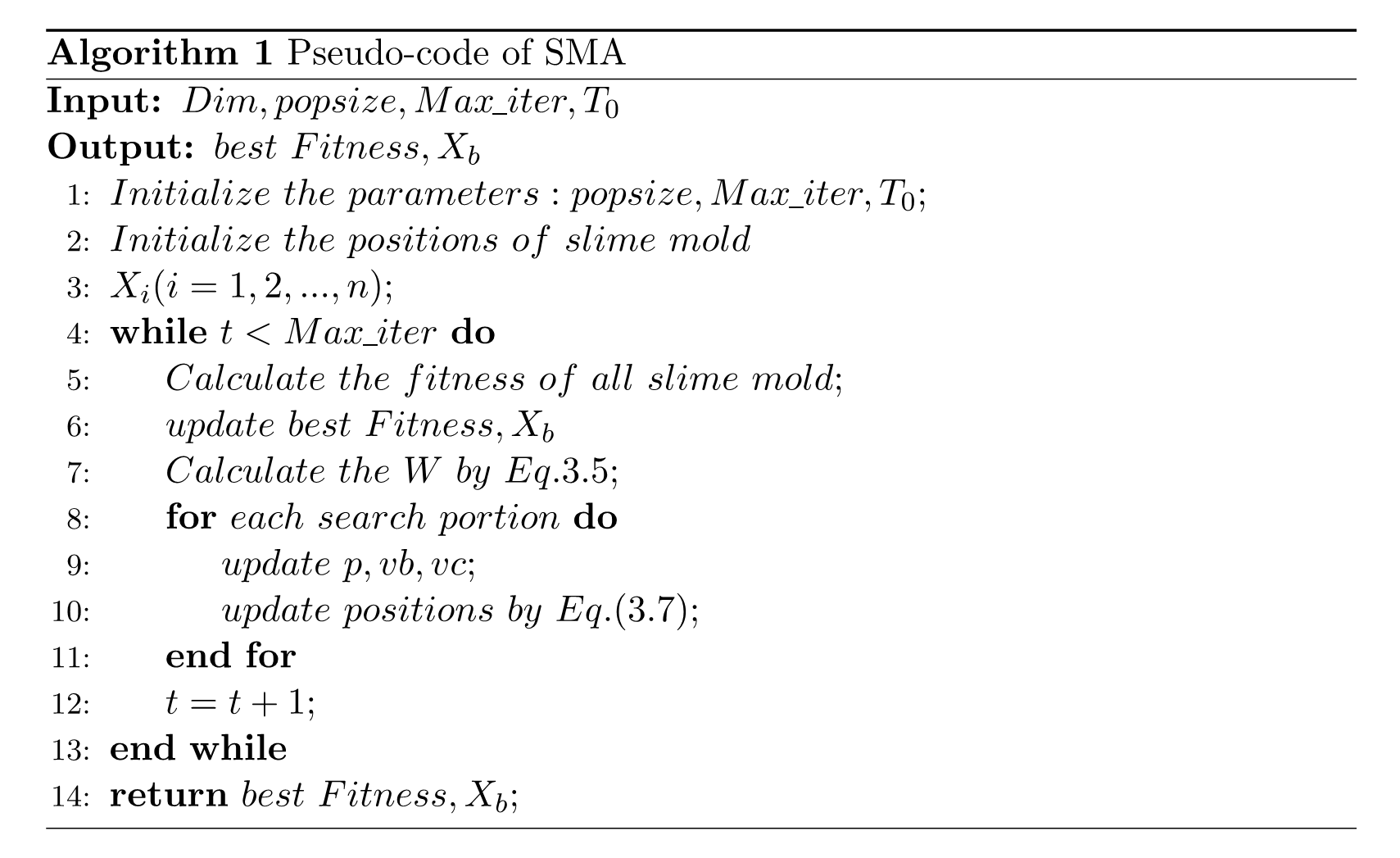

Supplement: S1 Algorithm — (TIF) [file pone.0280512.s001.tif]

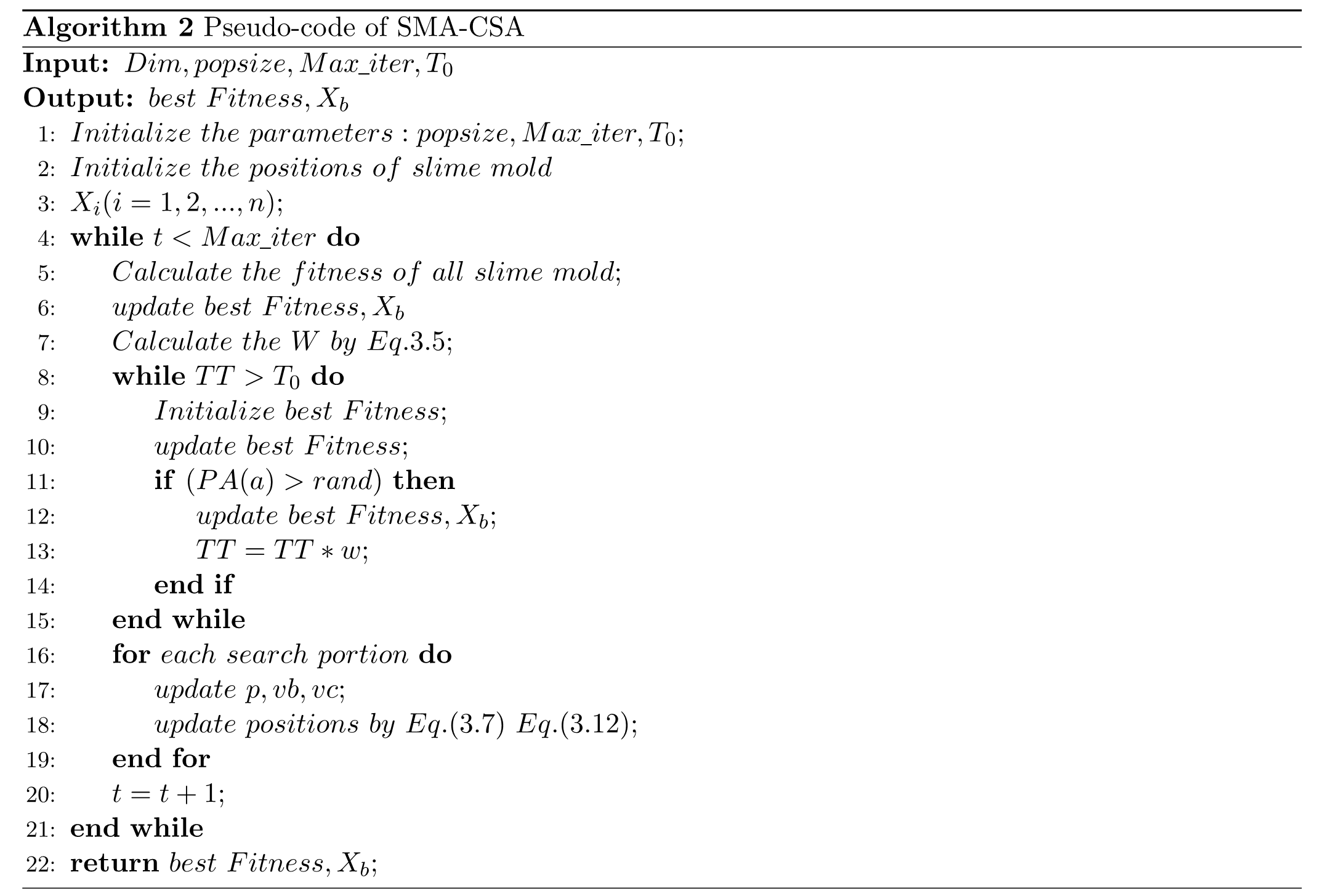

Supplement: S2 Algorithm — (TIF) [file pone.0280512.s002.tif]
