# Supplementary material for: Improved slime mould algorithm based on hybrid strategy optimization of Cauchy mutation and simulated annealing
Source: PLoS One. 2023 Jan 25;18(1):e0280512. doi: 10.1371/journal.pone.0280512 (PMC9876378; doi:10.1371/journal.pone.0280512)
Supplement: S1 Appendix — (DOCX) [file pone.0280512.s003.docx]

**Appendix**

Part of the comparison algorithm that is not marked with the full name in the text can be obtained from the following:

**AOA:** Archimedes optimization algorithm

**TSA:** Tunicate Swarm Algorithm

**GWO:** Grey Wolf Optimizer

**ESMA:** Equilibrium slime mould algorithm

**L-SHADE:** Successful History-based Adaptive DE variants with linear population size reduction

**CMA-ES:** The CMA Evolution Strategy

**CSMA:** Chaotic slime mould optimization algorithm

**ILS-RVND:** Hybrid algorithm based on Iterated Local Search (ILS) and Random Variable Neighborhood Descent (RVND) metaheuristics

**ISOS:** Improved Symbiotic Organisms Search

**EACO:** An enhanced ant colony optimization

**LNS-ACO:** Hybrid algorithm that executes large neighborhood search algorithm in combination with the solution construction mechanism of the ant colony optimization algorithm

**GRELS:** A GRASP × Evolutionary Local Search Hybrid

**AGES:** Active-guided evolution strategies

**HGPSO:** A hybrid particle swarm optimization algorithm
